# Parasites revive hope for cancer therapy

**DOI:** 10.1186/s40001-024-02057-2

**Published:** 2024-10-05

**Authors:** Maha M. Eissa, Ahmed Ebada Salem, Nahla El Skhawy

**Affiliations:** 1 Department of Medical Parasitology, Faculty of Medicine, Alexandria University, Alexandria, Egypt; 2 Department of Radiology and Nuclear Medicine, School of Medicine, Huntsman Cancer Institute, University of Utah, Salt Lake City, UT 48123 USA

**Keywords:** Cancer immunotherapy, Cancer vaccine candidates, Parasitic immunotherapy, Immunomodulation, Anti-angiogenesis, Molecular mimicry theory

## Abstract

Parasites have attained a life-long stigma of being detrimental organisms with deleterious outcomes. Yet, recently, a creditable twist was verified that can dramatically change our perception of those parasites from being a source of misery to millions of people to a useful anti-cancerous tool. Various parasites have shown promise to combat cancer in different experimental models, including colorectal, lung, and breast cancers, among others. Helminths and protozoan parasites, as well as their derivatives such as *Echinococcus granulosus* protein KI-1, *Toxoplasma gondii* GRA15II, and *Trypanosoma cruzi* calreticulin, have demonstrated the ability to inhibit tumor growth, angiogenesis, and metastasis. This article provides an overview of the literature on various cancer types that have shown promising responses to parasite therapy in both in vitro and in vivo animal studies. Parasites have shown anti-neoplastic activity through a variety of mechanisms that collectively contribute to their anti-cancer properties. These include immunomodulation, inhibition of angiogenesis, and molecular mimicry with cancer cells. This review article sheds light on this intriguing emerging field and emphasizes the value of collaborative multidisciplinary research projects with funding agencies and pharmaceutical companies. Thus, these strategies would secure continuous exploration of this new avenue and accelerate the advancement of cancer therapy research. Although experimental studies are heavily conducted by leaps and bounds, further steps are definitely lagging. Upgrading research from the experimental level to the clinical trial would be a wise progression toward efficient exploitation of the anti-neoplastic capabilities of parasites, ultimately saving countless lives.

## Introduction

Cancer is a worldwide life-threatening concern that imposes heavy health and economic burdens every year. Approximately 20 million new cancer cases and 10 million deaths are reported annually [1]. By 2040, it is expected that the global burden of new cancer cases will increase to 30 million [2]. This emphasizes the value of exploring new therapeutic horizons to chase safe, efficient drugs and detour away from traditional cancer treatments such as chemotherapy, radiotherapy, and surgery that inevitably have lethal drawbacks [3]. Recent advancements in cancer therapeutic and preventive methods include immunotherapy, targeted therapy, precision medicine, and gene therapy [4].

Cancer immunotherapy is a promising approach to fighting cancer by restoring the balance of the immune system. It is an emerging and revolutionary weapon with various successful trials, offering hope in the battle against cancer [5]. Advancements in cancer immunotherapy have been significant every decade. It effectively targets cancer cells by engaging the immune system creating biological stimulation to break the tolerance and eliciting a hostile environment for tumor growth**.** Multiple categories are included under the umbrella of cancer immunotherapy, such as adoptive T-cell transfer, immune checkpoint inhibitors (negative regulators of T cells), cytokine therapies, and cancer vaccines [6]. Aside from the immunomodulatory agents in cancer pipeline immunotherapeutic trials, pathogens have evolved as promising candidates [7].

Recently, pathogens and their products have been included within the framework of cancer immunotherapy by referring to the hygiene hypothesis [8]. It attributes immune-related diseases such as autoimmune and allergic diseases to a lack of exposure to different microorganisms during childhood [9]. This theory has been re-evaluated and stated cancer as another outcome of the hygiene hypothesis, explaining the relevant use of pathogens and their products to fight cancer. Therefore, pathogens and their products have been included within the framework of cancer immunotherapy [10].

Cancer vaccines, a wide category in cancer immunotherapy, can be industrialized either by utilizing cancer antigens, immune cells, pathogens, or their products to excite the immune system [7, 11]. One of the main challenges in the development of an efficient anti-cancer vaccine is the limited ability of tumor-associated antigens to overcome immune tolerance [12]. This can be explained by the nature of cancer cells and their antigens being familiar and not foreign to the patient’s body, thus eliciting a weak antigenic immune response if any. In addition, cancer cells have evoked various tools to evade the immune response [13]. On the contrary, pathogens are foreign to the host and are more easily recognized by the immune cells than cancer cells, and thus can elicit a stronger immune response [14]. Therefore, a wide range of pathogens and their products, including viruses and bacteria, have been added to the growing list of cancer immunotherapy trials to stimulate the immune response [10]. For instance, the usage of the human papilloma virus (HPV) vaccine [15] and hepatitis B virus (HBV) vaccine [16] as preventive cancer vaccines that can preclude cervical cancer and hepatocellular carcinoma, respectively. Bacillus Calmette–Guérin (BCG) is a type of therapeutic cancer vaccine. BCG vaccine harboring attenuated *Mycobacterium bovis*; a prophylactic tuberculosis vaccine is now repurposed as a therapeutic vaccine against bladder cancer [11] and metastatic melanoma [17].

Parasites are harmful organisms that can cause debilitating diseases, within which some are approved biological carcinogens. *Schistosoma haematobium* (*S. haematobium*) infection is known to cause bladder cancer, and *Clonorchis sinensis* (*C. sinensis*) and *Opisthorchis viverrini* (*O. viverrini*) are associated with cholangiocarcinoma [18]. However, the immunomodulatory benefits of parasites cannot be ignored and have drawn attention as talented immunomodulatory and anti-neoplastic armamentariums [14, 19]. Numerous studies have revealed their potential benefits for various autoimmune diseases, including arthritis [20], and bronchial asthma [21], and ulcerative colitis [22] among others [23, 24]. For cancer, different research groups reported an adverse relationship between certain parasitic infections and cancer in the human population. Lower cancer prevalence has been observed in patients with hydatid cyst disease or chagasic megacolon compared to the normal population [25, 26]. Likewise, a negative correlation was found between malaria incidence and cancer mortality [27]. Additionally, a state of resistance to cancer was observed in individuals with a low titer of anti-*Toxoplasma* antibody [28]. This was in line with the other epidemiological data that reported longer survival rates of cancer patients; simultaneously infected with certain parasites than non-infected patients [29, 30].

## Antineoplastic mechanistic strategies of parasites

**Fig. 1:**
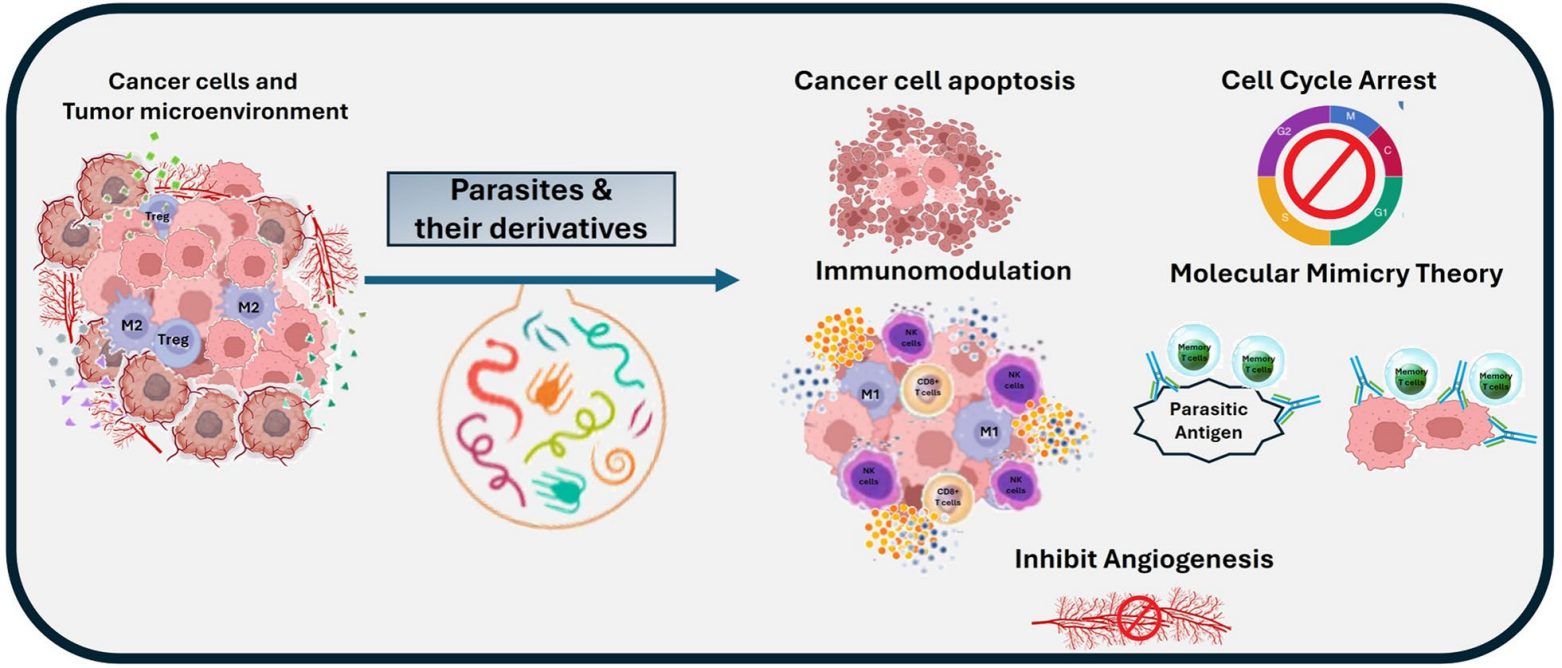
Diagrammatic illustration of the variuos anti-neoplastic mechanistic strategies of parasites

Relying on multi-mechanistic strategies, parasites have successfully promoted anti-neoplastic potency in various experimental cancer models [10, 19]. Immunomodulation, inhibition of angiogenesis, and the molecular mimicry theory are the three main cornerstones that undoubtedly build the anti-neoplastic power of parasites (Fig. 1) [31–35].

Cancer has recently been included in the re-evaluated hygiene hypothesis illustrating that the lack of exposure to pathogens accelerates and promotes cancer development [10, 35]. As a consequence of the persistence of long-term parasitic infections along with the release of their by-products, the host’s immune system is in a continuous specific immune-stimulatory state to combat the parasitic infection, especially with their highly provoked immune-evasive strategies [32, 35]. Thus, this can be exploited as an extremely valuable credit to stimulate the hijacked immune system within the suppressed cancer microenvironment [35]. Initially, at the molecular level, parasites promote an increase in co-stimulatory molecules expression. This initiates a cycle of Th 1 immune stimulation including both innate and adaptive immune cells [36]. Via restoring an imbalanced immune system, parasites and their antigens were able to stimulate the MyD88 pathway and release robust pro-inflammatory cytokines such as INF-γ, TNF-α, IL-1, IL-2, IL-12, and others, thus creating an immunostimulant environment that is hostile to tumor cells. These factors promote the differentiation of macrophages to M1 form, thus stimulating dendritic cells (DCs) and activating NK cells and T helper cells toward Th 1 responses [10, 14, 19, 32, 37]. The proliferation of parasite-specific immune cells such as eosinophils within some parasitic infections is another cancer-combating strategy [35]. Additionally, parasites suppress IL-10 and tumor growth factor (TGF) expression [34, 38]. At the level of cytotoxic T cells, parasitic infections stimulate CD8^+^ T cells to harbor more granzymes and perforin, thus amplifying their cytotoxic potency. Eventually, parasites promote tumor-selective cytotoxic T-cell invasion and suppress T regulatory (Treg) cell recruitment to the tumor tissue. The efficient rise in CD8^+^ T-cell/Treg ratio is a vital prognostic factor that has been efficiently achieved by parasites [36, 39–41].

Furthermore, the influence of parasites on the induction of apoptotic pathways was also reported. It is interesting to note that certain parasite molecules trigger apoptosis only in cancer cells while leaving normal cells unaffected. For instance, the *T. cruzi* surface molecule gp82 has been found to induce apoptosis in melanoma cells without impacting normal melanocytes [42]. Additionally, the use of hydatid cyst fluid has been shown to trigger apoptosis in various tumor cells [41].

Additionally, *T. spiralis* is one of those missionary parasites. Despite its potency in the induction of Th 2 immune response that conflicts with its usage as an anti-neoplastic agent, its excretory–secretory antigen; especially during the early phase of muscle invasion is different. Those antigens independently and efficiently induce mitochondrial apoptotic and death-receptor pathways such as Bax, and caspases, as well as activation of p53, signifying an anti-neoplastic potency. This is achieved in addition to the stimulation of immune cells, including macrophages, NK cells, and cytotoxic T cells, releasing INF-γ, TNF-α, and many other interleukins [43, 44]. Thus, identifying parasitic molecules that can selectively induce apoptosis in cancer cells could be a promising approach for cancer therapy. This would allow for targeting cancer cells without causing harm to normal ones.

Along with the novel emerging era of the use of checkpoint inhibitors in cancer immunotherapy to unleash the activity of the immune cells, interestingly some parasites, such as *Toxoplasma* and *Plasmodium* effectively inhibited programmed cell death and its ligand (PD-1/PDL-1). Thus, they simultaneously work as a checkpoint inhibitor, unleashing the power of T-cell activity, especially on CD8^+^ T cells [11, 36, 45].

Angiogenesis is an essential factor in tumor growth. Failure to achieve this factor declares tumor cell death. Through a direct strategy, parasites suppress vascular development within tumor tissue via suppression of vascular endothelial growth factor (VEGF). Interestingly, via an indirect mechanism, VEGF has an immune-suppressor activity. Consequently, interfering with angiogenesis stimulates the immune system [46]. Thus, parasites can attack two birds with one stone, inhibit angiogenesis, and stimulate the suppressed immune system [35, 39, 40].

A unique criterion that discriminates parasites from other immunotherapeutic agents is the molecular mimicry theory [19, 47]. The sharing of antigenic epitopes between parasites and cancer cells has exceeded being a theory to a verified phenomenon with excellent experimental applications. Mucin-type *O*-glycan structures (Tn, Tk, sial Tn, TF) that are crucial cancer cell invasion and metastasis markers were documented to be expressed in many parasites including trematodes, cestodes, and protozoa as well [14, 48–50]. The use of these heterogenic purified shared antigens separately induced anti-neoplastic activity in an experimental breast cancer model in mice [51] denoting their ability to break immune tolerance and demonstrate good immunogenicity. Therefore, these parasites and their antigens could offer a new direction for cancer biotherapy. On the other hand, several studies have shown that anti-parasite antibodies could target those shared antigens bound to specific cancer cells [52, 53], induce their death, and reduce experimental tumor growth [54]. Therefore, anti-parasite antibodies that selectively react with cancer cells but not with normal cells may be potential candidates for targeted cancer therapy in humans. These results probably justify the occurrence of anti-neoplastic activity selectively between certain parasites and specific cancer cell lines and not others [14, 39, 55]. Hence, this phenomenon adds a privilege for the use of parasites and their products as favored anti-neoplastic candidates.

Thus, parasites use a multifactorial approach to combat cancer, using a variety of strategies, such as inhibiting proliferative signals, regulating inflammatory responses, promoting apoptosis, activating the immune response, preventing metastasis, and interfering with angiogenesis [19, 35].

Thus, researchers are currently heavily investigating certain parasites as potential allies in the battle against cancer. Experimental studies have successfully demonstrated the potency of certain parasites as cancer warriors. Therefore, this review article summarizes up-to-date literature conducted on various cancer types showing promising response to parasite therapy both in vitro and in vivo. The responsible mechanisms hypothesized to be involved in modulating cancer progression were also elaborated. The goal is to shed light on this intriguing emerging field and emphasize the importance of collaborative multidisciplinary research projects between different areas of science for the potential development of novel, highly immunogenic, and nature-based cancer therapeutics for incurable cancers.

## Database search

To examine the most relevant literature used in this review, we searched electronic databases including ScienceDirect, PubMed, Egyptian Knowledge Bank, Scopus, and Google Scholar. Search terms used in various combinations were “parasites, helminths, *Echinococcus granulosus*, *Trichinella spiralis*, protozoa, *Trypanosoma cruzi*, *Plasmodium*, *Toxoplasma gondii*, parasite antigens/antibodies/derivatives” and “anti-cancer, anti-neoplastic, anti-tumor, cancer immunotherapy”. Articles published up to January 2024 have been searched, and comprehensively reviewed, and their bibliographies were checked for other relevant publications. This review included experimental research conducted either in vitro or in vivo, for the results to be controlled and validated.

## Parasites’ anti-neoplastic activity against various cancers

Until January 2024, a total of 182 research articles have demonstrated the potency of a wide range of parasites and their derivatives as potent anti-neoplastic activity verified in both in vitro and in vivo. The anti-neoplastic response of parasites against different cancer types is presented and summarized in Tables 1, 2, 3, 4, 5, 6, 7, and 8.

**Table 1:** a and b: in vitro and in vivo studies of the anti-neoplastic activity of different parasites against gastrointestinal cancers (colorectal cancer, gastric and esophageal cancers)

a. Colorectal carcinoma			
Parasite	Type of study	Antineoplastic activity	References
*Schistosoma mansoni*			
Autoclaved *S. mansoni* cercarial antigen	In vivo	Inhibited colon carcinogenesis as demonstrated by a notable reduction in the size and number of neoplasms per mouse and induced immunomodulatory potential	[55]
*Echinococcus granulosus*			
Hydatid cyst fluid	In vitro	Induced apoptosis in C26 mouse colon cancer cells	[71]
Anti-hydatid cyst fluid antibodies	Flow cytometry	Reacted with antigens on CT26 mouse cancer colon cells	[72]
Human hydatid cyst fluid	In vivo	Protected against colon cancer growth both prophylactically and therapeutically	[72]
Hydatid cyst fluid, 78 kDa fraction, and live protoscolices	In vivo	Significantly inhibited colon cancer growth	[73]
*Taenia solium*			
Recombinant *T. solium* Calreticulin (r*Ts*CRT) alone and in combination with 5-fluorouracil	In vitro	Inhibited colon cancer cell growth (SW480 cell line) in a dose-dependent manner	[75]
*Taenia crassiceps*			
*T. crassiceps* infection	In vivo	Inhibited the colonic inflammatory responses and CRC formation	[78]
*Trichinella spiralis*			
*T. spiralis* infection	In vivo	Inhibited HCT-8 human colorectal carcinoma growth as demonstrated by a decrease in size and weight of tumors in BALB/c mice	[80]
*Toxocara canis*			
*T. canis*-derived peptides [fraction from the excretory–secretory Troponin protein peptide (ES TPP)]	In vitro	Induced cellular changes, decreased cellular viability, and significantly altered expression of gene (Bcl-2, APAF1, ZEB1, VEGF, cyclin-D1, and caspase-3) in colon carcinoma cell lines (HT-29, and Caco2) in a dose-dependent manner	[82]
*Heligmosomoides polygyrus*			
*H. polygyrus* adult*-*derived antigen and excretory/secretory products (HES)	In vitro	Significantly reduced the proliferation of CT26.WT murine colorectal cancer cells HES alone significantly reduced HCT116 human colorectal cancer cell proliferation	[61]
*Trypanosoma cruzi*			
*T. cruzi* infection	In vivo	Low incidence of chemically induced colon cancer in rats	[87]
*T. cruzi e*pimastigote lysates	In vivo	Strongly inhibited colon cancer development in a rat model and evoked an integrated anti-tumor response involving both the cellular and humoral components of the immune response	[52]
Anti-*T. cruzi* polyclonal antibodies	Immunohistochemistry	Cross-reacted with human colon cancer cells	[52]
*Plasmodium* spp.			
Infection with *Plasmodium yoelii* 17XNL-infected erythrocytes	In vivo	Reduced weight and size of tumors by inhibiting proliferation and promoting mitochondria-mediated apoptosis in colon cancer cells	[89]
Recombinant malarial protein VAR2 CSA as a carrier for anti-cancer drug delivery, VDC886 [hemiasterlin analog (KT886) conjugated rVAR2]	In vitro	Strong cytotoxicity to human colon cancer cells (Colo 205)	[90]
Recombinant malaria’s sporozoite circumsporozoite protein	In vitro	Inhibited the proliferation of the human colon cancer cell line, SW480 in a dose-dependent manner and induced apoptosis by suppressing the activation of the NF-κB	[91]
*Toxoplasma gondii*			
GRA16, a dense granule protein of *T. gondii*	In vitro	Induce apoptosis of HCT116 human colorectal cancer cells by down-regulation of the expression of human telomerase reverse transcriptase	[93]
Recombinant dense granular protein GRA8 of *T. gondii* in conjugation with the acidity-triggered rational membrane(ATRAM) [rATRAM-GRA8-M/AS]	In vitro and in vivo	Induced cell death of HCT116 human colorectal cancer cells in a dose-dependent manner and significantly inhibited tumor growth in HCT116 xenograft animal model	[94]
Attenuated RH-ΔGRA17 *T. gondii* strain in combination with programmed death ligand-1 (PD-L1) blockade	In vivo	Combination therapy significantly haltered tumor growth compared to each treatment alone, increased lymphocyte infiltration, and sensitized the tumor microenvironment in local and distal tumors to PD-L1 blockade	[95]
*T. gondii* lysate antigen	In vivo	Reduced tumor growth in CT26 colorectal carcinoma euthymic and athymic murine models	[96]
*T. gondii*, profilin-like protein (*Tg*PLP) as an adjuvant to autologous whole-tumor cell vaccine treatment	In vivo	Enhanced anti-tumor immune responses of autologous whole-tumor cell vaccine as demonstrated by a smaller tumor in CT26 colorectal carcinoma murine model and increased their survival	[97]
*T. gondii* rGRA6Nt protein as an adjuvant to non-replicable MC38 colorectal cancer cells immunization	In vivo	Significantly inhibited the growth of the implanted tumors and increased CD8^+^ infiltration within the tumor microenvironment in a murine model of MC38 CRC	[98]
Exosomes derived from *T. gondii*-infected dendritic cells (Me49-DC-Exo)	In vivo	Significantly slowed the tumor growth, inhibited macrophage polarization to M2 phenotype, and regulated the suppressor of cytokine signaling 1 SOCS1 expression by delivering functional miR-155-5p in a mouse model of CRC	[100]
Exosomes derived from dendritic cells infected with *T. gondii* (DC-Me49-Exo)	In vivo	Significantly inhibited tumor growth by reducing the proportion of myeloid-derived suppressor cells (MDSCs) in a mouse model of CRC	[99]
*Eimeria stiedae*			
*E. stiedae* oocyst soluble protein	In vivo	Enhanced the anti-tumor immune response, inhibited tumor growth, lung metastasis, and angiogenesis in the murine model of colon cancer (CT26)	[102]

b. Gastric and esophageal carcinomas			
Parasite	Type of study	Antineoplastic activity	References
*Trichinella spiralis*			
Crude *T. spiralis* extract from adult worms and newborn larvae	In vivo	Inhibited tumor growth in mice grafted with murine forestomach carcinoma (cell line MFC)	[104]
*Toxocara canis*			
*T. canis*-derived peptides [synthesized peptide fraction from the excretory–secretory Troponin protein peptide (ES TPP)]	In vitro	Induced cellular changes, decreased cellular viability, and significantly altered the cellular expression of gene (BCL-2, APAF1, ZEB1, VEGF, cyclin-D1, and caspase-3) in a gastric cell line (AGS) in a dose-dependent manner	[82]
*Toxoplasma gondii*			
*T. gondii* tachyzoites	In vitro	Significant inhibitory effect on human esophageal squamous cell carcinoma cell line EC109 proliferation	[105]
*T. gondii* culture supernatant	In vitro	Inhibited the proliferation and induced apoptosis in human gastric cancer BGC-823 cells in a concentration- and time-dependent manner	[106]
Live *T. gondii or T. gondii* lysate	In vitro	Reduction of the multidrug resistance of human gastric cancer cell line against cytostatic drugs	[107]

**Table 2:** a and b: in vitro and in vivo studies of the anti-neoplastic activity of different parasites against hepato-pancreatic cancers (hepatocellular carcinoma and pancreatic cancer)

a. Hepatocellular carcinoma			
Parasite	Type of study	Antineoplastic activity	References
*Schistosoma japonicum*			
*S. japonicum* Sja-miRNA-7-5p	In vitro and in vivo	Significantly inhibited the migration of mouse and human hepatoma cells and significantly inhibited tumor growth in a xenograft animal model through down-regulation of the S-phase kinase-associated protein 2 gene	[111]
*S. japonicum* Sja-miR-3096	In vitro and in vivo	Significantly inhibited the migration of both murine and human hepatoma cell lines and significantly inhibited tumor growth in the murine model by targeting phosphoinositide 3-kinase class II alpha (PIK3C2A)	[112]
*S. japonicum* Sja-miR-61	In vitro and in vivo	Significantly inhibited the migration of both mouse and human hepatoma cells and significantly inhibited tumor growth in the murine model through anti-angiogenesis activity by targeting the PGAM1 gene	[113]
*S. japonicum* Sja-miR-71a	In vitro and in vivo	Significantly inhibited the migration of both mouse and human hepatoma and human HCC cell lines and significantly inhibited tumor growth in a xenograft mouse model by targeting the *FZD4* gene	[114]
*Fasciola hepatica*			
*F. hepatica* (biologically active substances)	In vitro	Significant inhibitory effect on MC29 hepatoma cell line proliferation	[116]
*Trichinella spiralis*			
*T. spiralis* crude antigens (mixture from adult and newborn larvae)	In vitro	Apoptosis of ascitic hepatoma (cell line H22), and hepatoma cell line (H7402)	[104]
*T. spiralis* Excretory secretory products (ESP) from adult worms or muscle larvae	In vitro	Inhibited cellular proliferation and induced apoptosis in liver cancer (H22) cells through the mitochondrial pathway	[117]
*T. spiralis* infection	In vivo	Significantly inhibited mouse ascitic hepatoma H22 in ICR mice	[117]
*T. spiralis* protein encoded by the A200711 gene	In vitro	Induced apoptosis in human hepatoma H7402 cells	[118]
*T. spiralis* larval extract (peptide2 matched the hypothetical protein T01_4238 of *T. spiralis*)	In vitro	Inhibition of proliferation of HepG2 cells in a dose-dependent manner	[119]
*T. spiralis* infection	In vivo	Significantly inhibited hepatoma Hep1-6 carcinoma growth in the C57BL/6 mice	[120]
*T. spiralis* infection	In vivo	Decreased progression of the tumor with increased rate of apoptosis as shown by the decreased expression of Bcl-2 and increased rat survival time	[121]
*Angiostrongylus cantonensis*			
*A. cantonensis*, excretory–secretory products (ESPs) and recombinant Calreticulin (rCRT) (a key effector protein of ESPs)	In vitro	Both induced apoptosis in human hepatoma HepG2 cells. rCRT had a stronger inhibitory effect on HepG2 cells compared to ESPs	[123]
*A. cantonensis*, *excretory–secretory* products (ESPs)	In vivo	Significantly reduced tumor growth in Hepa1-6 mouse tumor model	[123]
*Setaria equina*			
*S. equina* excretory–secretory products (*Se*ES) alone and in combination with diethylcarbamazine citrate (DEC)	In vivo	Had a beneficial effect on HCC development in the rat model by increasing the activity of antioxidant enzymes and decreasing the expression of NF κB. Combination with DEC modulates the antioxidant effect of *Se*ES and induces immunomodulatory and protective effects on rat HCC	[124, 125]
Rabbit anti-*S. equina* extract and diethylcarbamazine citrate polyclonal IgG antibodies	Indirect ELISA and Western blotting	Cross-reactive protein bands in *S. equina* and Huh-7 hepatoma cells at 75 and 70 kDa by anti-*S. equina* and anti-diethylcarbamazine antibodies, respectively	[126]
*Plasmodium* spp.			
*P. berghei* infection	In vivo	Reduced hepatic carcinoma development induced by aflatoxin B1 in rats	[128]
*P. yoelii 17 XNL infection*	In vivo	Significantly inhibited tumor progression in murine implanted hepatoma model and prolonged their survival time through suppression of angiogenesis within the tumor microenvironment	[129]
*Plasmodium*-based vector (*P*.*y*-GPC3) *P.* yoelii 17XNL expressing murine glypican-3 protein (expressed in Hepa1-6 cells)	In vivo	Significantly inhibited Hepa1-6-induced tumor growth in the implanted HCC murine model, prolonged their survival time, and induced tumor antigen-specific T-cell-mediated immunity	[131]
*P. yoelii* infection	In vivo	Significantly inhibited the recurrence and metastasis and improved the prognosis of HCC in both non-resection and resection murine orthotopic HCC models	[132]
*Toxoplasma gondii*			
*T. gondii* tachyzoites RH strain	In vitro	Inhibited the proliferation and induced apoptosis of HCC (H7402) cells in a concentration-dependent manner	[133]
GRA16, a dense granule protein of* T. gondii*	In vitro and in vivo	Decreased cell proliferation, anti-apoptotic factors, p‐AKT/AKT ratio, cell migration, and invasive activity of hepatocellular carcinoma (HepG2) cells and decreased tumor size in HepG2 cell‐xenograft nude mice	[134]
*T. gondii* GRA15II-polarized macrophages	In vitro and in vivo	Significantly inhibited migration and invasion of the Hepa1-6 cells and markedly restricted tumor growth in tumor-bearing C57BL/6 mice	[135]

b. Pancreatic cancer			
Parasite	Type of study	Antineoplastic activity	References
*Echinococcus granulosus*			
*E. granulosus* live protoscolex to induce secondary hydatidosis	In vivo	Significantly inhibited the development of precursor foci of neoplastic changes in the pancreas in the azaserine-rat model	[138]
*Toxoplasma gondii*			
A live, non-replicating avirulent uracil auxotroph vaccine strain (cps) of *T. gondii*	In vivo	Significantly regressed established tumor in pancreatic ductal adenocarcinoma mouse model	[139]
A live, non-replicating avirulent uracil auxotroph vaccine strain (cps) of *T. gondii*	In vivo	Induced significant therapeutic benefit through generating anti-tumor immune responses in established aggressive disseminated pancreatic cancer in a mouse model	[140]
A live, non-replicating avirulent uracil auxotroph vaccine strain (cps) of* T. gondii*	In vivo	Stimulated long-term strong immune protection against the recurrence of pancreatic cancer in mice that survived the primary pancreatic tumor after cps treatment	[141]
Attenuated *Toxoplasma* NRTUA strain and combination therapy with anti-PD-1 antibody	In vivo	Monotherapy with attenuated *Toxoplasma* NRTUA inhibited tumor growth in a mouse model of PDAC and its combination with anti-PD1 antibody enhanced its anti-tumor activity and controlled tumor growth in Pan02 tumor-bearing mice	[142]
*T. gondii* soluble antigen and *T. gondii* profilin	In vivo	Significantly decreased tumor volume and increased tumor infiltration with CD4^+^ and CD8^+^ T cells	[143]

**Table 3:** In vitro and in vivo studies of the anti-neoplastic activity of different parasites against lung cancer

Parasite	Type of study	Antineoplastic activity	References
*Echinococcus granulosus*			
Human hydatid cyst fluid	In vivo	Induced strong anti-tumor immune response that significantly inhibited LLC growth both in prophylactic and therapeutic settings and significantly increased mouse survival	[147]
Hydatid cyst fluid	Immunoelectrophoresis	Reacted with the serum from a patient with pulmonary carcinoma forming a broad and intense precipitin band	[148]
Sera of patients with hydatid diseases	In vitro	Cytotoxic effects on NCI-H209/An1 lung cancer cells	[149]
*Trichinella spiralis*			
*T. spiralis* infection	In vivo	Inhibited tumor growth which is dependent on the time of tumor challenge to *T. spirals* infection	[150]
*T. spiralis* muscle-larva excretory–secretory proteins	In vitro	Inhibited proliferation and induced apoptosis in small-cell lung cancer cells (H446 SCLC) and non-small-cell lung cancer line A549 in a dose and time-dependent manner	[151–153]
*T. spiralis* recombinant small Heat Shock Proteins (sHSP)	Western immunoblotting	Reacted with anti-LLC antiserum	[154]
*T. spiralis* small Heat Shock Protein (sHSP) gene using invasive *Lactobacillus plantarum* (NC8) as vector vaccine (NC8-sHSP)	In vivo	Inhibited tumor growth in both prophylactic and therapeutic experiments through induction of humoral, cellular, and mucosal immunity	[155]
*Trichomonas vaginalis*			
*T. vaginalis* trophozoites	In vitro	Elicited cytotoxic effect and induced apoptosis in human lung alveolar basal carcinoma epithelial cell line A549	[158]
*Trypanosoma* spp*.*			
*T. cruzi* epimastigote-derived protein lysate	In vivo	Prevented tumor growth in murine lung cancer model through induction of both humoral and cellular anti-tumor immune responses in therapeutic and preventive settings	[157]
*T. brucei* hypoxanthine–guanine phosphoribosyltransferase (TbHGPRT)	In vitro	Had cytotoxic effects and induced apoptosis in one murine lung cancer cell line (M27) and four human non-small cell lung carcinoma cell lines (A549, H460, H661, H520)	[163]
*Plasmodium* spp*.*			
*Plasmodium* infection alone and in combination with a lung cancer DNA vaccine [pcDNA3.1-hMUC1]	In vivo	Inhibited the growth and metastasis of lung cancer and increased the survival of tumor-bearing mice and when combined with lung cancer DNA vaccine it boosted the immune response and had a synergistic anti-tumor effect	[164]
*Plasmodium*-infected red blood cells	In vivo	Reduced Treg and myeloid-derived suppressor cells and elevated CD8^+^ T-cell-mediated cytotoxicity within the tumor microenvironment	[165]
Exosomes derived from the plasma of *Plasmodium-*infected mice	In vivo	Significantly reduced LLC growth in mice	[160]
Genetically attenuated *Plasmodium* sporozoites	In vivo	Inhibited lung cancer growth and angiogenesis	[166]
Attenuated *Plasmodium* sporozoite as a novel vector to lung cancer-specific antigen [MAGE-A3]	In vivo	Inhibited tumor growth, increased mice survival rate, and induced a strong MAGE-A3-specific CD8^+^ T-cell response	[167]
*Plasmodium* circumsporozoite protein overexpression	Flow cytometry, colony analysis, laser confocal microscopy assay and western blotting	Increased sensitivity of A549 and H1975 cancer cell lines to gefitinib as demonstrated by significant apoptosis and decreased viability of cells and significant inhibition of autophagy	[168]
*Plasmodium* circumsporozoite protein overexpression	In vivo	Synergistically inhibited cancer growth by enhancing the chemotherapeutic effect of gefitinib by inhibition of autophagy	[168]
*Plasmodium* circumsporozoite protein	In vitro	Inhibited the proliferation of the human lung cancer cell line, A549 in a dose-dependent manner and induced apoptosis by suppressing the activation of NF-κB	[169]
*Plasmodium yoelii*-infected red blood cells	In vivo	Inhibited tumor growth and progression in LLC mouse model	[170]
*P. yoelii* infection combined with radiotherapy	In vivo	Induced synergistic anti-tumor effects in a subcutaneous model of mouse non-small cell lung cancer (LLC, CRL-1642) and significantly inhibited tumor growth and extended the life span of tumor-bearing mice	[171]
*P. chabaudi* infection alone and in combination with Gemcitabine treatment	In vivo	Significantly inhibited tumor growth, metastasis, and prolonged mice survival in a subcutaneous implanted murine LLC model, and combination therapy was more effective than monotherapy	[172]
*Toxoplasma gondii*			
*T. gondii* tachyzoites	In vitro	Significant inhibitory effect and induced apoptosis in non-small-cell lung cancer cells (cell line A549) in a dose-dependent manner with up-regulation of p53 and down-regulation of Bcl-2 gene expression	[105]
Formalin-fixed *T. gondii* organisms	In vivo	Potent anti-tumor effects against LLC	[159]
*T. gondii* Me49 strain infection	In vivo	Inhibited LLC growth through induction of Th1 immune responses and inhibition of angiogenesis	[174]
*T. gondii* excretory–secretory antigen	In vivo	Inhibited tumor growth in LLC-bearing mice and reduced the proportion of splenic Treg cells in splenocytes	[175]
GRA16, a dense granule protein of *T. gondii* alone and in combination with the anti-cancer irinotecan	In vivo	Reduced tumor size in a xenograft mouse model of non-small-cell lung carcinoma with synergistic promotion of the anti-cancer effects of Irinotecan	[176]
Attenuated RH-ΔGRA17 *T. gondii* strain in combination with programmed death ligand-1(PD-L1) blockade	In vivo	Combination therapy reduced tumor size compared to each treatment alone with up-regulated innate and adaptive immune responses, increased lymphocyte infiltration, and sensitized the tumor microenvironment in local and distal tumors to PD-L1 blockade treatment	[95]

**Table 4:** In vitro and in vivo studies of the anti-neoplastic activity of different parasites against breast cancer

Parasite	Type of study	Antineoplastic activity	References
*Echinococcus granulosus*			
Hydatid cyst fluid antigens such as antigen B, glycolipid, glycoprotein, and 78 kDa fractions	In vitro	Induced apoptosis in mouse breast cancer cells (4T1 cell line)	[179]
Infection with hydatid cyst	In vivo	Reduced the development of breast cancer tumors by 50% in DMBA-induced breast cancer rat model	[180]
Hydatid cyst wall antigens	Western immunoblotting	A considerable cross-reactivity with breast cancer patients’ sera at a specific band (~ 27/28 kDa)	[51]
Hydatid cyst wall antigen (the protein bands with cross-reactivity with breast cancer patients’ sera (~ 27/28 kDa)	In vivo	Significant inhibition of 4T1 breast tumor growth in murine mammary carcinoma model	[51]
Hydatid cyst wall antigen antigens, including crude extract of HCW (laminated layer) 28 and 27 kDa protein bands (upper and lower bands)	In vivo	Significantly Inhibited breast tumor growth and metastasis and improved survival in 4T1 tumor-bearing mice	[181]
Hydatid cyst fluid and antigen B	In vivo	Significantly reduced breast tumor size with polarization of the Th1/Th2 ratio toward the Th1 pathway	[182]
Recombinant Kunitz-type protease inhibitor EgKI-1 from *E. granulosus*	In vitro and in vivo	Inhibited the growth and migration of breast cancer cell lines (MCF-7, T47D, and MDA-MB-231) in a dose-dependent manner and induced significant tumor growth reduction in triple-negative breast cancer (MDA-MB-231) murine model	[186]
Rabbit antisera raised against hydatid cyst antigens (hydatid cyst fluid, germinal and laminated, protoscolex, and excretory–secretory)	In vitro	Induced cell death of 4T1 breast cancer cell line	[187]
Rabbit antisera raised against hydatid cyst wall antigen	Flow cytometry	A significant high % of 4T1 breast cancer cell reaction	[51]
Rabbit antisera raised against hydatid cyst antigens (protoscolex, cell wall, and cystic fluid antigens)	Flow cytometry	Reacted with the breast cancer cell line (4T1)	[188]
Hydatid cyst antigens (hydatid fluid, laminated and germinal layer antigens, and excretory–secretory antigens of protoscolices) and rabbit antisera raised against laminated and germinal layers of hydatid cyst	Western immunoblotting	A reaction between hydatid cyst antigens and sera of patients with breast cancers. Antisera raised against laminated and germinal layers of hydatid cyst reacted with excretory–secretory products of breast cancer cells	[48]
*Taenia solium*			
Recombinant *T. solium* Calreticulin (r*Ts*CRT) either alone or in combination with 5-fluorouracil	In vitro	Inhibited growth of breast (MCF-7) cancer cells by interacting with scavenger receptors	[75]
*Trichinella spiralis*			
*T. spiralis* infection	In vivo	Increased survival rate in mice breast tumor	[189]
*T. spiralis* infection	In vivo	Inhibited mammary gland cancer	[190]
*Setaria equina*			
Rabbit anti-*S. equina* extract *and* diethylcarbamazine citrate polyclonal IgG antibodies	Indirect ELISA and Western blotting	Cross-reactive protein bands in *S. equina* and MCF-7 cells at 75 and 70 kDa by anti-*S. equina* and anti-diethylcarbamazine antibodies respectively	[126]
*Leishmania* spp*.*			
Live attenuated *L. infantum* and *L. tropica*	In vivo	Both *Leishmania* species induced regression of 4T1 breast cancer in mice after intratumoral injection	[191]
*Trypanosoma cruzi*			
*T. cruzi* epimastigote lysate	In vitro	Inhibition of breast cancer cell growth	[192]
Recombinant *T. cruzi* Calreticulin (*rTc*CRT)	In vivo	Antiangiogenic and anti-tumor properties inhibited the growth of the murine mammary tumor (TA3 MTXR tumor cell line)	[194]
Rat anti-*T. cruzi* polyclonal antibodies	In vitro, flow cytometry, and Immunohistochemical analysis	Reacted with human mammary cancer cells (T47D and MCF-7 tumor cell lines) and capable of mediating antibody-dependent cellular cytotoxicity	[52]
*T. cruzi* epimastigote lysates	In vivo	Protective immune response against breast cancer	[52]
*T. cruzi infection and* epimastigote extract	In vivo	Inhibited the growth of an aggressive mammary adenocarcinoma cell line (TA3-MTXR) in mice and identified that nTcCRT is responsible for the anti-tumor activity	[195]
*T. cruzi* recombinant protein 21 (rP21)	In vitro	Reduced migration, invasion, and progression in triple-negative breast cancer cells MDA-MB-231 cells	[197]
*T. cruzi* infection	In vitro	Inhibited proliferation and migration of triple-negative breast cancer cell line (MDA-MB-231)	[199]
*Plasmodium* spp*.*			
Recombinant malarial VAR2 CSA as a carrier for anti-cancer drug delivery, VDC886 [hemiasterlin analog (KT886) conjugated rVAR2]	In vitro and in vivo	*S*trong cytotoxicity to human breast cancer cells (MDA-MB-231 and 4T1 breast cancer cell lines) and increased survival of mice with 4T1 with no metastasis as compared to control mice, all of which died of metastatic disease	[90]
*P. yoelii infection*	In vivo	Significantly inhibited tumor growth, and increased the survival rate of the tumor-bearing mice in a murine triple-negative breast cancer model (4T1)	[200]
*Toxoplasma gondii*			
*T. gondii* tachyzoites	In vitro	Significant inhibitory effect on MCF-7 breast cancer cell line proliferation in a dose-dependent manner	[105]
*T. gondii* tachyzoites	In vitro	Marked destruction of Her2/Neu-expressing mammary cancer cell lines (TUBO cells) in a dose- and time-dependent manner	[201]
*T. gondii* tachyzoites	In vitro	Marked destruction of Her2/Neu-expressing mammary cancer cell lines (TUBO cells) in a dose- and time-dependent manner	[202]
Rabbit anti-*T. gondii* antibodies	Flow cytometry	Reacted with breast cancer cells (4T1 cell line)	[53]
Human anti-*Toxoplasma* antibodies	Flow cytometry	Strongly reacted with breast cancer cells (4T1 and MCF-7 cell lines)	[203]
*T. gondii* chronic infection	In vivo	Significant resistance to the development of mammary tumors in C_3_H/_He_ mice	[204]
Attenuated uracil auxotroph strain of *T. gondii*	In vivo	Intratumoral inoculation of this attenuated *Toxoplasma* strain inhibited tumor growth, and metastasis, increased survival of the 4T1 tumor-bearing mice, and increased the secretion of IL-12 and INF-γ in both the serum and tumor microenvironment	[205]
*Neospora caninum*			
*N. caninum* tachyzoites	In vitro	Invaded and multiplied within human breast carcinoma cells (MCF-7 cell line) leading to their destruction	[208]
*Besnoitia jellisoni*			
*B. jellisoni* chronic infection	In vivo	Significant resistance to the development of mammary tumors in C_3_H/_He_ mice	[204]

**Table 5:** a–d: in vitro and in vivo studies of the anti-neoplastic activity of different parasites against hematological cancers (leukemia, lymphomas, multiple myeloma, and mastocytoma)

a. Leukemia			
Parasite	Type of study	Antineoplastic activity	References
*Schistosoma japonicum*			
*S. japonicum* recombinant protein (r*Sj*16)	In vitro	Significantly suppressed the proliferation of murine myeloid leukemia WEHI-3B JCS cells in a dose and time-dependent manner by inducing G0/G1 cell cycle arrest and apoptosis via activation of the caspase-mediated mechanism by regulating the expression of Bcl-2 family	[38, 211]
*Echinococcus granulosus*			
Protoscolex hydatid cyst somatic antigens	In vitro	Induced programmed cell death in human chronic myeloid leukemia cell line K562 cells in a dose and time-dependent manner	[212]
*Trichinella spiralis*			
*T. spiralis* crude antigens	In vitro	Inhibited proliferation and induced apoptosis of human chronic myeloid leukemia cell line K562 cells	[104]
*T. spiralis* excretory/secretory (ES) products from muscle larvae	In vitro	Induced cell death with high apoptotic index 72 h following treatment of acute myeloid leukemia cell line (U937 cells)	[214]
Peritoneal macrophages from *T. spiralis-*infected mice one week post-infection	In vitro	Strong cytotoxic effect on the R1 leukemia cells	[215]
*Marshallagia marshalli*			
*M. marshalli* somatic antigen	In vitro	Inhibited proliferation and induced apoptosis in human chronic myeloid leukemia K562 cells	[216]
*Trypanosoma cruzi*			
Rabbit polyclonal antibodies against *T. cruzi*	Confocal microscopy assay	Showed the presence of antigenic proteins by immunodetection in acute lymphoblastic leukemia cells with antibodies against *T. cruzi*	[217]
*Plasmodium* spp*.*			
Recombinant malarial protein VAR2 CSA as a carrier for anti-cancer drug delivery, VDC886 [hemiasterlin analog (KT886) conjugated rVAR2]	In vitro	Induced strong cytotoxicity to myeloid leukemia cells (KG-1 cell line)	[90]
*P. yoelii* infection	In vivo	Significantly reduced the growth of murine WEHI-3 leukemia cells in mice, marked decrease in tumor cell infiltration into the liver and spleen, and elicited anti-leukemia activity by promoting immune response	[218]
*Toxoplasma gondii*			
*T. gondii* tachyzoites	In vitro	Inhibited proliferation and induced apoptosis in human chronic myeloid leukemia K562 cells	[213]
Chronic *T. gondii* infection	In vivo	Induced resistance to the development of leukemia in AKR mice	[204]
*Besnoitia jellisoni*			
*B. jellisoni* chronic infection	In vivo	Induced resistance to the development of leukemia in AKR mice	[204]

b. Lymphoma			
Parasite	Type of study	Antineoplastic activity	References
*Trichinella spiralis*			
*T. spiralis* activated macrophages from *T. spiralis-*infected mice	In vitro	Induced powerful cytotoxic effect on syngeneic EL-4 lymphoma cells	[222]
*Trypanosoma cruzi*			
*T. cruzi* infection	In vivo	Inhibited growth and metastasis of solid L5178Y-R lymphoma transplanted to BALB/c mice due to the production of cytotoxic/cytostatic substances and cross antibodies against both the parasite and the tumor antigens	[223]
*Plasmodium* spp*.*			
Recombinant malarial protein VAR2 CSA as a carrier for anti-cancer drug delivery, VDC886 [hemiasterlin analog (KT886) conjugated rVAR2]	In vitro and in vivo	Induced strong cytotoxicity to MyLa2059 T-cell lymphoma and significantly inhibited the growth of non-Hodgkin’s lymphoma (Karpas 299) in the murine model	[90]
*Toxoplasma gondii*			
Formalin-fixed *T. gondii* organisms	In vivo	Induced a potent anti-tumor effect and a significant suppression of tumor growth	[224]
*T. gondii* lysate	In vivo	Enhanced the cytotoxicity of feline spleen cells against feline leukemia virus-producing lymphoma cells	[225]
*T. gondii* lysate antigen, spleen cells from *Toxoplasma* lysate antigen sensitized mice	In vivo and in vitro	Inhibited tumor growth induced by 20-methylcholanthrene in BALB/c mice and induced cytolytic effect on YAC-1 murine lymphoma cell line	[226]
Live *T. gondii* or *T. gondii* lysate	In vitro	Reduction of the multidrug resistance of mouse lymphoma cell lines (Mdr L 5718 and Par 5718) against cytostatic drugs	[107]

c. Multiple myeloma			
Parasite	Type of study	Antineoplastic activity	References
*Fasciola hepatica*			
*F. hepatica* (biologically active substances)	In vitro	Slight inhibitory effect on myeloma cell culture proliferation	[116]
*Trichinella spiralis*			
*T. spiralis* antigen and positive sera	Enzyme-linked immunosorbent assays (ELISA)	Cross reaction between antigens of the myeloma cell SP2/0 and positive sera to *T. spiralis*, and antigens of *T. spiralis* versus positive sera to myeloma cell SP2/0	[231]
Antisera against a novel gene product of *T. spiralis*	Immunoscreened a *T. spiralis* cDNA expression library	Cross-reacted with Sp2/0 myeloma cells	[232]
*T. spiralis* infection	In vivo	Suppression-subtractive hybridization identified genes encoding RpL41, NKTR, Rbbp4, and AN-XA2 as possible proteins involved in tumor growth inhibition in SP2/0 myeloma model BALB/c mice	[233]

d. Mastocytoma (mast cell tumor)			
Parasite	Type of study	Antineoplastic activity	References
*Trichinella spiralis*			
Activated macrophages from *T. spiralis-*infected mice	In vitro	Inhibitory effect on allogeneic P815 mastocytoma cells	[222]
*Toxoplasma gondii*			
*T. gondii* lysate antigen	In vitro	Strong cytolytic activity on P-815, a murine mastocytoma cell line	[226]

**Table 6:** a and b: in vitro and in vivo studies of the anti-neoplastic activity of different parasites against connective tissue and bone cancers (sarcomas and bone tumors)

a. Sarcomas			
Parasite	Type of study	Antineoplastic activity	References
*Schistosoma mansoni*			
*S. mansoni* infection	In vivo	Induced resistance against a transplantable sarcoma 180 ascites tumor	[241]
*Echinococcus granulosus*			
*E. granulosus* live protoscolices	In vitro	Inhibited proliferation and induced cell death in WEHI-164 fibrosarcoma Cells	[242]
*Trichinella spiralis*			
*T. spiralis* crude antigens	In vitro	Anti-proliferative activity on sarcoma cells (cell line S180)	[104]
*T. spiralis* infection	In vivo	Modify the host reaction to S-180 that is dependent on the time of tumor inoculation post-infection and prolonged the survival time	[243]
*T. spiralis* infection	In vivo	Inhibited tumor growth under selected conditions of larval dose and tumor cells challenge interval	[244]
*T. spiralis* infection, crude extracts of adult and newborn larvae	In vivo	Inhibited tumor growth in a dose-dependent manner	[104]
*Toxocara canis*			
*T. canis* egg antigens	In vivo	Inhibited WEHI-164 fibrosarcoma growth in BALB/c mouse model	[245]
*Leishmania* spp*.*			
*L. donovani* sphingolipid-1	In vitro and in vivo	Induced significant cytotoxic effects on murine sarcoma 180 cells in vitro via alteration of mitochondrial pathway. Improved the survival rate of tumor-bearing mice and reduced tumor growth in a sarcoma 180 murine model	[247, 248]
*Toxoplasma gondii*			
*T. gondii* infection	In vivo	Induced resistance to the development of sarcoma 180 in Swiss mice	[204]
*T. gondii* antigen	In vivo	Inhibited WEHI-164 fibrosarcoma growth in BALB/c mouse model	[245]
Dendritic cells treated with protein components of *T. gondii*	In vivo	Induced tumor infiltration with CD8^+^ T cells and improved the survival of the mice in a mouse model of fibrosarcoma (WEHI-164)	[249]
Dendritic cells treated with protein components of *T. gondii*	In vivo	Inhibited tumor growth and increased activity of cytotoxic T-cell-mediated anti-tumor response in a mouse model of fibrosarcoma (WEHI-164)	[250]
*T. gondii* lysate antigen	In vivo	Reduced tumor size and reduced the expression of CD31, an angiogenesis marker in the tumor tissue in sarcoma 180 mouse model	[251]
Live *T. gondii* tachyzoites	In vitro	Decreased fibrosarcoma WEHI-164 cell proliferation and increased cell lyses	[252]
*Eimeria tenella*			
Soluble protein extract of *E. tenella* sporulated oocysts (native or recombinant protein)	In vivo	Induced potent immunostimulatory effects and significantly reduced tumor growth, prolonged survival time, and cure rates of above 80% in the murine model of sarcoma (S-180)	[253]
*Besnoitia jellisoni*			
*B. jellisoni* infection	In vivo	Significant resistance to the development of sarcoma 180 in Swiss mice	[204]

b. Bone tumors (malignant: osteosarcoma, Ewing sarcoma, chondrosarcoma and other malignant tumors and Benign tumors: osteochondroma, giant tumor, exostosis, and other benign tumor)			
Parasite	Type of study	Antineoplastic activity	References
*Echinococcus granulosus*			
*E. granulosus*, crude extract of the laminated layer antigens (43 kDa and 70 kDa)	Western blot	Both antigens reacted with the serum of different bone cancers	[256]
*Trichinella spiralis*			
Anti-*T. spirals* antiserum and anti-TPD52 antiserum (prepared against cross-antigen between *T. spiralis* and osteosarcoma)	In vitro and in vivo	Inhibited proliferation of human osteosarcoma MG-63 cells and induced apoptosis in a dose-dependent manner. In the osteosarcoma murine model, both anti-*T. spiralis* antiserum and anti-TPD52 antiserum inhibited osteosarcoma growth by inducing apoptosis and enhancing immunity without causing histopathological damage but anti-TPD52 antiserum was more effective than anti-*T. spiralis* antiserum	[54]

**Table 7:** a and b: in vitro and in vivo studies of the anti-neoplastic activity of different parasites against skin cancers (melanoma and Merkel-cell carcinoma)

a. Melanoma			
Parasite	Type of study	Antineoplastic activity	References
*Echinococcus granulosus*			
Hydatid cyst fluid, fraction of hydatid cyst fluid, alive protoscolices or their excretory–secretory antigen	In vivo	Inhibited melanoma cancer growth in C57/black mice	[261–263]
Hydatid cyst fluid (fertile/infertile)	In vitro	Induced apoptosis on A375 human melanoma cancer cells	[264]
Antigen B from hydatid cyst fluid	In vitro	Inhibited proliferation and promoted apoptosis of B16F10 melanoma cancer cell line	[265]
Recombinant Kunitz-type protease inhibitor EgKI-1 from *E. granulosus*	In vitro	Inhibited the growth and migration of melanoma (CJM) cells in a dose-dependent manner through disruption of the cell cycle and induction of apoptosis	[186]
*Taenia crassiceps*			
GK-1, an 18-amino acid peptide from *T. crassiceps* cysticerci either alone or in combination with anti-programmed death ligand 1	In vivo	Administration of GK1 as monotherapy induced significant prolongation of survival time of melanoma-bearing mice, delayed tumor development with increased tumor necrosis. Combination therapy induced significant prolongation of melanoma-bearing mice survival time compared to monotherapy with GK1 alone and decreased TNF-α, IL-4, IL-5, IL-6, and IL-10 serum levels	[266, 267]
*Trichinella spiralis*			
*T. spiralis* infection	In vivo	No detectable tumor in infected mice	[268]
*T. spiralis* infection	In vivo	Reduced tumor growth and lung metastasis of B16–F10 melanoma cells	[269]
*T. spiralis* chronic infection	In vivo	Reduced tumor size	[270]
*T. spiralis* chronic infection	In vivo	Inhibited tumor development, longer tumor induction interval with significant reduction in tumor size	[271]
*T spiralis* excretory–secretory antigen of the first larval stage	In vitro	Induced apoptosis in B16 melanoma cells	[270]
*Leishmania* spp*.*			
*L. donovani* sphingolipid-1	In vitro and in vivo	It has cytotoxic effects and induces apoptosis in murine and human melanoma cells (B16F10 and A375 cell lines). In murine melanoma model, it induced improvement of survival time, immunostimulatory response, significant reduction in rumor volume, and reduced the metastatic flare of angiogenic factors	[247, 272, 273]
*Acanthamoeba castellanii*			
Viable *A. castellanii and* parasite lysates	In vitro and in vivo	Induced extensive cytolysis of human melanoma (OCM-1) and murine melanoma (D5.1G4) cells and intratumoral injection led to a reduction in tumor mass	[275]
*Trypanosoma cruzi*			
*T. cruzi* surface molecule gp82 (a recombinant protein J18)	In vitro and in vivo	Induced cell death and apoptosis in melanoma cells and reduced melanoma tumor size in C57BL/6 mice	[42]
*T. cruzi* calreticulin in combination with plasmid encoding survivin, a tumor-associated antigen	In vivo	Induced as synergistic inhibitory effect on tumor growth in a B16-F10 melanoma murine mode	[276]
*T. cruzi* derived Toll-Like Receptor (TLR) agonists (glycoinositolphospholipids and CpGs oligodeoxynucleotides)	In vivo	Induced significant protective immunity and cause a significant delay in the growth of B16F10 melanoma cells expressing NY-ESO-1 in a murine model	[277]
A recombinant non-pathogenic clone of *T. cruzi* as a vaccine vector expressing a cancer-testis antigen (NY-ESO-1)	In vivo	Induced strong and long-term T-cell-mediated immunity and solid protection against melanoma in mice	[278]
Combination of non-pathogenic clone of *T. cruzi* expressing a cancer-testis antigen (NY-ESO-1) with blockade of Cytotoxic T Lymphocyte Antigen 4	In vivo	Induced significant therapeutic response and controlled the development of an established melanoma in the murine model	[279]
*Plasmodium* spp*.*			
*P. yoelii* infection (17XL strains)	In vivo	Delayed occurrence of tumor and inhibited the growth of melanoma in B16F10 melanoma-bearing mice	[280]
Recombinant malarial protein VAR2 CSA as a carrier for anti-cancer drug delivery, VDC886 [hemiasterlin analog (KT886) conjugated to rVAR2]	In vitro	Induced strong cytotoxicity to C32 human and B16 murine melanoma cells	[90]
Recombinant malaria protein VAR2 CSA-malaria mimicking erythrocyte nanoparticles loaded with the anti-neoplastic agent, docetaxel	In vitro and in vivo	Killed melanoma cells in vitro and effective targeting and therapeutic efficacy in a xenografted melanoma model	[281]
Recombinant malaria protein VAR2 CSA-modified activated platelet-mimicking nanoparticles (rVAR2-PM/PLGA-ss-HA) loaded with the anti-neoplastic agent, docetaxel	In vivo	Exhibited a significant synergistic and active tumor-targeting, anti-neoplastic effect against primary and lung metastasis in a mouse model of melanoma (B16F10)	[282]
*Toxoplasma gondii*			
*T. gondii* infection	In vivo	A significant suppression of melanoma growth and strong systemic suppression of angiogenesis	[283]
Excreted/secreted antigens of *T. gondii*	In vivo	Suppressed tumor growth by down-regulation of CD4^+^CD25^+^ Treg cells while up-regulation of NK cells	[284]
*T. gondii* tachyzoites antigens (fraction A1 and C14-dendritic cells)	In vivo	A1 matured dendritic cells are more potent than C14 to induce immune response leading to slowing tumor growth and prolonged survival time in the melanoma mouse mode	[285]
Non-replicating uracil auxotroph strain of *T. gondii* (cps strain)	In vivo	Effectively haltered tumor growth, stimulated systemic anti-tumor immunity, and modified tumor microenvironment	[286]
Attenuated *T. gondii* mutants lacking two lactate dehydrogenases (ME49 *Δldh1–Δldh2*)	In vivo	Intratumoral administration of *Δldh1-Δldh2* repressed the growth of established tumors and helped to inhibit lethal tumor development in the mouse melanoma (B16F1) model	[287]
Attenuated RH-ΔGRA17 *T. gondii* strain in combination with programmed death ligand-1 (PD-L1) blockade	In vivo	Combination therapy arrested tumor growth compared to each treatment alone with up-regulation of innate and adaptive immune pathways and sensitized the tumor microenvironment in local and distal tumors to PD-L1 blockade	[95]
Rabbit anti-*Toxoplasma* antibodies	Flow cytometry	Reacted strongly with mouse melanoma cancer cells	[53]
*Neospora caninum*			
*N. caninum* tachyzoites	In vivo	Significant inhibition of tumor growth, significant increase in the mRNA expression levels of IL-12, IFN-γ, IL-2, IL-10, TNF-α, and PD-L1 in the tumor microenvironment, and IL-12 and IFN-γ in the spleen of tumor-bearing mice and increased infiltration of CD8^+^ T cells and macrophages in the tumor microenvironment	[288]
Wild *N. caninum* and engineered *N. caninum secreting* IL-15/IL 15Rα	In vivo	Engineered *N. caninum* was more potent than wild *N. caninum* in impairing lung metastasis progression and induced a marked increase in natural killer cells, CD8^+^ T cells, and macrophages polarization toward M1 Phenotype in lung tissue of syngeneic C57BL6 mouse model of B16-F10 melanoma lung metastases	[289]
*Babesia microti*			
*B. microti* infection	In vivo	Inhibited the growth of melanoma in tumor-bearing mice and prolonged their survival time through activation of immune responses	[291]

b. Merkel-cell carcinoma			
Parasite	Type of study	Antineoplastic activity	References
*Neospora caninum*			
*N. caninum* tachyzoites	In vitro and in vivo	They infected and destroyed Merkel-cell carcinoma [The WaGa (RRID:CVCL E998)]. They triggered potent anti-tumor immune response leading to a threefold reduction in tumor size in a model of NOD/SCID mice-bearing human Merkel-cell carcinoma	[294]

**Table 8:** a–e: in vitro and in vivo studies of the anti-neoplastic activity of different parasites against miscellaneous cancers (gynecological cancers, prostate cancer, neuroglial and neural crest cancers, thymoma, and head and neck squamous cell carcinoma)

a. Gynecological cancers			
a.1. Ovarian cancer			
Parasite	Type of study	Antineoplastic activity	References
*Taenia solium*			
Recombinant *T. solium* Calreticulin (rTsCRT) either alone or in combination with 5-fluorouracil	In vitro	Inhibition of the growth of ovarian cancer cells (SKOV3 cell line) in a dose-dependent manner	[75]
*Toxoplasma gondii*			
Nonreplicating uracil auxotroph strain of *Toxoplasma* (cps strain)	In vivo	Induced therapeutic anti-tumor immunity through reversed immunosuppression in the tumor microenvironment	[300]
Secretion of rhoptry and dense granule effector proteins by non-replicating *T. gondii* uracil auxotrophs strain	In vivo	Induced anti-tumor immunity through host responses involving CD8^+^, α dendritic cells, the IL-12/IFN-γ Th 1 axis, as well as CD4^+^ and CD8^+^ T cells and reverting immunosuppression in the ovarian cancer microenvironment	[301]
*T. gondii* extract	In vitro	Inhibited proliferation of susceptible (A2780) and resistant (A2780-CP) ovarian cancer cell lines to cisplatin	[302]
*Leishmania* spp*.*			
*L. major extract*	In vitro	Inhibited proliferation of susceptible (A2780) and resistant (A2780-CP) ovarian cancer cell lines to cisplatin	[302]

a.2. Cervical cancer			
Parasite	Type of study	Antineoplastic activity	References
*Echinococcus granulosus*			
Recombinant Kunitz-type protease inhibitor EgKI-1 from *E. granulosus*	In vitro	Inhibited the growth and migration of cervical epithelial adenocarcinoma HeLa cells in a dose-dependent manner through disruption of the cell cycle and triggering of apoptosis	[186]
*Taenia solium*			
Recombinant *T. solium* Calreticulin (r*Ts*CRT) either alone or in combination with 5-fluorouracil	In vitro	Inhibited the growth of cervical HeLa cancer cell line in a dose-dependent manner through interaction with scavenger receptors	[75]
Hookworm *Nippostrongylus brasiliensis*			
*N. brasiliensis* L3 antigen	In vitro	Reduced cervical cancer cell migration in the following lines [HeLa (HPV18-positive epithelial adenocarcinoma cells), Ca Ski (HPV16 positive epithelial epidermoid carcinoma cells), and C33-A (HPV negative epithelial carcinoma cells)] and significantly reduced the expression of vimentin and N-cadherin, and decreased HPV type16 pseudovirion internalization	[61]
*N. brasiliensis* infection	In vivo	Significant reduction of vimentin expression within the female genital tract in the murine model	[61]
*Trichinella spiralis*			
Biologically active substances isolated from the livers of *T. spiralis-infected* rats	In vitro	Induced a mild inhibiting effect on the growth of human cervical carcinoma HeLa cells by inhibition of proliferation and promotion of apoptosis	[304]
*Trichomonas vaginalis*			
Culture supernatant of *T. vaginalis*	In vitro	Inhibited the growth of HeLa cervical cancer cells by inhibition of proliferation and promotion of apoptosis	[305]
Live *T. vaginalis* and excretory–secretory product	In vitro	Induced apoptosis in human cervical mucosal epithelial SiHa cells through the dissociation of Bcl-xL/Bim and Mcl-1/Bim complexes	[306]
Live *T. vaginalis*	In vitro	Induced apoptosis in human cervical mucosal epithelial SiHa cells by parasite dose-dependent reactive oxygen species production through an NF-*κ*B-regulated, mitochondria-mediated pathway	[307]
Live *T. vaginalis and T. vaginalis* excretory and secretory product	In vitro	Live parasite-induced cytotoxicity and mitochondrial reactive oxygen species production in human cervical mucosal epithelial SiHa cells in a parasite burden and infection time-dependent manner *T. vaginalis* excretory/secretory product induced apoptosis through mitochondria reactive oxygen species and endoplasmic reticulum stress responses, and also promoted endoplasmic reticulum stress-mediated mitochondrial apoptosis via the IRE1/ASK1/JNK/Bcl-2 family protein pathways	[308]

b. Prostate cancer			
Parasite	Type of study	Antineoplastic activity	References
*Trichomonas vaginalis*			
Culture supernatant of *T. vaginalis*	In vitro	Inhibited proliferation and promoted apoptosis of PC-3 and DU145 prostate cancer cell lines cells, through up-regulation of p21 and down-regulation of Bcl-2	[312]
*Plasmodium* spp*.*			
Recombinant malarial rVAR2-DT fusion protein and VDC886 [hemiasterlin analog (KT886) conjugated rVAR2]	In vitro and in vivo	Induced strong cytotoxicity to human prostate cancer cells (PC-3) and significantly inhibited the growth of prostate cancer (CP-3) in a mouse model	[90]
*Toxoplasma gondii*			
*T. gondii* tachyzoites	In vitro	Significant inhibitory effect on DU-145 prostate cancer cell line proliferation	[105]

c. Neuroglial and neural crest cancer			
c. 1. Glioma			
Parasite	Type of study	Antineoplastic activity	References
*Trichinella spiralis*			
*T. spiralis* infection (different muscle larvae doses)	In vivo	Induced a parasite load-dependent anti-tumor effect	[315]
*Trichomonas vaginalis*			
*T. vaginalis* purine nucleoside phosphorylase (*Tv*PNP) as an activating enzyme for F-araAMP or deoxyadenosine analogs cancer therapy	In vivo	Excellent anti-tumor activity in *Tv*PNP/D54 glioma-bearing tumors in mice	[316]
*Plasmodium* spp*.*			
Recombinant malarial protein VAR2 CSA as a carrier for anti-cancer drug delivery, VDC886 [hemiasterlin analog (KT886) conjugated rVAR2]	In vitro	Induced strong cytotoxicity to glioma cells	[90]
*P. yoelii* infection alone and combined with radiotherapy	In vivo	Induced synergistic anti-tumor effects in orthotopic and subcutaneous models of mouse glioma (GL261). The combination therapy significantly slowed tumor growth and extended the life span of tumor-bearing mice more than monotherapy. Most importantly, the combination therapy could cure approximately 70 percent of gliomas	[171]
*Toxoplasma gondii*			
*Toxoplasma* lysate antigen alone and in combination with Quil-A	In vitro and in vivo	*S*uppressed proliferation and invasion of human glioma U373MG and U87MG cells in a dose-dependent manner and reduced tumor growth in athymic nude mice transplanted with human malignant glioma cells. The addition of Quil-A significantly enhanced its anti-tumor effects	[317]

c.2. Medulloblastoma			
Parasite	Type of study	Antineoplastic activity	References
*Toxoplasma gondii*			
*T. gondii* infection	In vivo	Reduced tumor incidence and transformed the non-supportive tumor microenvironment into a supportive one for T cells	[319]

c.3. Ependymoblastoma			
Parasite	Type of study	Antineoplastic activity	References
*Toxoplasma gondii*			
*T. gondii* infection	In vivo	Significant reduction in tumor growth with strong inflammatory response surrounding the tumor with more necrosis in a mouse model of ependymoblastoma	[322]
Peritoneal macrophages from *Toxoplasma-*infected mice	In vitro	Cytotoxic to ependymoblastoma cells	[322]

c.4. Neuroblastoma			
Parasite	Type of study	Antineoplastic activity	References
*Trypanosoma cruzi*			
Rabbit polyclonal antibodies against *T. cruzi*	Confocal microscopy assay	Showed the presence of antigenic proteins by immunodetection in neuroblastoma cells reacted with antibodies against *T. cruzi*	[217]

d. Thymoma			
Parasite	Type of study	Antineoplastic activity	References
*Toxoplasma gondii*			
*T. gondii* (RH) strain tachyzoites	In vivo	In the murine thymoma EG7 mouse model, intratumoral inoculation of *T. gondii* tachyzoites induced strong inhibition of tumor development and total clearance of tumors in some mice within 25 days post-treatment	[294]
*Neospora caninum*			
*N. caninum* tachyzoites and engineered *N. caninum* strain (NC1-IL15hRec)	In vitro and in vivo	In vitro, *N. caninum* tachyzoites successfully infected and multiplied inside murine thymoma cells (cell line EG7) and caused their lysis. In murine thymoma EG7 mouse model, intratumoral or distal inoculation of *N. caninum* tachyzoites induced strong anti-tumor immunity and significantly inhibited tumor growth, often leading to complete eradication. Engineered *N. caninum* significantly enhanced its immunological properties	[294]

e. Head and neck squamous cell carcinoma			
Parasite	Type of study	Antineoplastic activity	References
*Trichomonas vaginalis*			
*T. vaginalis* purine nucleoside phosphorylase (*Tv*PNP) as an activating enzyme for deoxyadenosine analogs cancer therapy	In vivo	Exhibited robust tumor regressions in human head and neck squamous cell carcinoma (FaDu*/Tv*PNP) xenografts in nude mice	[316]

### Gastrointestinal cancers

Table 1a, b shows the in vitro and in vivo studies of the anti-neoplastic activity of different parasites against gastrointestinal cancers (Colorectal, Gastric, and Esophageal cancers).

#### Colorectal cancer (CRC)

Globally, colorectal cancer (CRC) is the third most commonly diagnosed cancer and the second leading cause of cancer-related deaths [56]. An effective therapy still does not exist, which necessitates an in-depth exploration of promising cancer immunotherapeutic modalities. Being an inflammatory-driven malignancy, the CRC microenvironment plays a crucial role in the behavior of the disease through the involvement of innate immunity. On the other hand, adaptive immunity has been found to play a role in cancer immunosurveillance [57].

#### Antineoplastic activity of helminth parasites against CRC

Despite the multiple factors that are listed to impose CRC, schistosomiasis is still a query-predisposing factor. Schistosomiasis is a neglected tropical parasitic disease that affects approximately 200 million people in the world’s tropical areas [58]. While there is conflicting evidence regarding the association between schistosomiasis and colon carcinogenesis in humans and experimental animals, the association between CRC and *Schistosoma mansoni* (*S. mansoni*) specifically is unjustified [59]. Interestingly, *S. mansoni* soluble egg antigen regulated the host’s immune system and achieved immunomodulation in an experimentally induced colitis model. It induced expansion of Foxp3^+^ Treg probably via an indirect antigen-presenting cells-mediated mechanism and direct effects on T cells. It attenuated experimentally trinitrobenzene sulfonic acid-induced colitis and protected mice from lethal inflammation [60]. Surprisingly, the soluble egg antigen of *S. mansoni*, which contains oncogenic antigens, was recently reported to have an unpredictable negative effect on colon cancer cell (CT26.WT) proliferation in vitro, even at high concentrations [61]. Interestingly, an earlier study in 1991 demonstrated the anti-neoplastic activity of *S. mansoni* antigen in a murine cancer model [62]. This is in agreement with a recent study by Eissa et al. [55] who reported a protective effect of the autoclaved *S. mansoni* cercarial antigen against chemically induced colon carcinogenesis in a murine model. It resulted in a notable reduction in the average size of lesions and the number of neoplasms per mouse. Additionally, schistosomal antigen provoked an immunomodulatory potential, as evidenced by a significant increase in serum interlukin-10 (IL-10), the percentage of splenic clusters of differentiation 4 T cells (CD4^+^ T cells), intestinal FoxP3^+^ Treg cells, and a significant decrease in serum IL-17 [55]. It is worth noting that in a series of previous studies, autoclaved parasitic antigens induced potent homologous protective immunity against experimental schistosomiasis [63], trichinellosis [64], toxoplasmosis [65], and leishmaniasis [66], as well as anti-cancer activity [39, 40, 55]. Their strong immunogenic potential was also capable of alleviating adjuvant-induced arthritis in a rat model [20] and has been demonstrated to be safe in both experimental studies and human clinical trials against leishmaniasis [67, 68]. These findings suggest that autoclaved parasitic antigens could be a promising, safe, and potent immunotherapeutic treatment. With advancements in technology, it will be possible to overcome challenges such as standardization or identification of the most antigenic components of these antigens. This could be a significant advancement in the pharmaceutical industry to develop nature-based therapeutics of parasitic origin that could target cancer, autoimmune diseases, and infectious diseases as well.

Another helminth parasite is *Echinococcus granulosus* (*E. granulosus*), the tapeworm that causes hydatid disease in humans; a cosmopolitan zoonotic disease [69]. It is transmitted by dogs or other canines, who act as definitive hosts, while herbivorous animals serve as intermediate hosts [70]. Humans can be sporadically infected and act as accidental intermediate hosts. Interestingly, there have been reports of anti-cancer effects associated with hydatid disease in humans [25, 29]. In an in vitro study, hydatid cyst fluid has been shown to exhibit anti-neoplastic activity by inducing apoptosis in a colon cancer cell line (C26) [71]. Moreover, vaccination with hydatid cyst fluid induced anti-neoplastic activity in a murine model, protecting against colon tumor growth in both prophylactic and therapeutic experimental trials [72]. Similarly, it was shown by Rostamirad et al. [73] that hydatid cyst antigens (hydatid cyst fluid, the 78 kDa fraction or live protoscolices); especially hydatid cyst fluid fraction significantly inhibited colon cancer tumor growth in BALB/c mice [73]. Furthermore, anti-hydatid cyst antibodies were able to identify cell surface and intracellular antigens in colon cancer cells (CT26) using flow cytometry. Proteomic analysis of colon cancer CT26 antigens cross-reacted with anti-hydatid cyst antibodies and hydatid cyst fluid identified two proteins: mortalin and creatine kinase M-type. Interestingly, CT26 mortalin showed 60% homology with *E. granulosus* hsp70 [72]. Thus, the anti-neoplastic activity of *E. granulosus* against CRC may be attributed to shared antigens and the development of an effective cross-reactive immune response against cancer cells.

*Taenia solium* (*T. solium*) is an intestinal tapeworm that is acquired by eating undercooked pork. Ingestion of pork meat that contains the larval stage, cysticercus cellulose, leads to human taeniasis. On the other hand, human cysticercosis/neurocysticercosis is a fecal–oral infection caused by ingesting eggs excreted in the feces of a human carrier of *T. solium* [74]. Interestingly**,** recombinant *T. solium* Calreticulin (r*Ts*CRT) was documented to promote an in vitro anti-tumoral effect. It inhibited the growth of the colon cancer cells (SW480 cell line) in a dose-dependent manner by interacting with scavenger receptors [75]. Although the main role of calreticulin, an endoplasmic reticulum protein, in *T. solium*, is to control intracellular Ca^+2^ homeostasis, it also participates in the assembly and surface expression of major histocompatibility complex (MHC) class I molecules for antigen presentation to CD8^+^ T cells, as well as in cell motility, secretion pathways, gene transcription regulation, protein synthesis, cellular adhesion, and apoptosis [76].

*Taenia crassiceps* (*T. crassiceps*) is another tapeworm that inhabits the intestine of carnivores, with rodents acting as its natural intermediate hosts. Human infection is rare and usually described in individuals with severe immunodeficiency. In such cases, humans act as an accidental intermediate host where the cysticerci, the larval stage of *T. crassiceps* can be found in skeletal muscles, subcutaneous tissue, or even intraocular [77]. The impact of *T. crassiceps* infection on the progression of colon cancer and colitis-associated colorectal tumorigenesis in mice was investigated. Infection with this mesocestod parasite inhibited the colonic inflammatory responses and CRC formation as well as prevented goblet cell loss. It also led to increased expression of IL-4, activated macrophage markers in colonic tissue, and negatively affected the expression of pro-inflammatory cytokines. Additionally, *T. crassiceps* infection prevented the up-regulation of β-catenin and CXCR2 expression, which are both markers associated with colitis-associated colorectal tumorigenesis [78].

*Trichinella spiralis* (*T. spiralis*), a tissue-dwelling helminth, causes human trichinellosis through ingestion of undercooked pork meat containing the parasite’s infective larvae. It possesses potent immunomodulatory properties and can stimulate anti-tumor immunity [79]. *Trichinella spiralis* infection has been shown to induce inhibition of HCT-8 colorectal carcinoma in BALB/c mice as demonstrated by a decrease in size and weight of tumors [80].

*Toxocara canis* (*T. canis*), a dog-specific nematode that can infect humans and cause visceral larva migrans and ocular larva migrans syndrome, has also shown an anti-neoplastic activity against colon cancer [81, 82]. *Toxocara canis-*derived peptides, specifically the synthesized peptide fraction from the excretory–secretory Troponin protein peptide, were able to induce cellular changes and decrease cellular viability in colon carcinoma cell lines (HT-29 and Caco2) in a dose-dependent manner and significantly altered the cellular expression of gene (Mcl-2, APAF1, ZEB1, VEGF, cyclin-D1, and caspase-3) involved in cancer progression [82].

In another study, derived antigens as well as excretory/secretory products (ES) from adult *Heligmosomoides polygyrus* (*H. polygyrus*), a gastrointestinal helminth parasite of rodents, significantly reduced the in vitro proliferation of murine (CT26.WT) colorectal cancer cells. *Heligmosomoides* excretory/secretory products (HES) alone are also significantly reduced the in vitro proliferation of human (HCT116) colorectal cancer cells, potentially through an increase in cell cycle arrest rather than cell apoptosis [61].

#### Antineoplastic activity of protozoan parasites against CRC

Apart from helminths, *Trypanosoma cruzi* (*T. cruzi*), a flagellated protozoan parasite that causes Chagas’ disease in humans possesses anti-cancer activities. This finding was first reported by Russian researchers Roskin and Exempliarskaia in 1931 who observed the tropism of *T. cruzi* toward tumor cells [83]. Later on, the anti-cancer activity of *T. cruzi* was further reported in several studies [84–86]. The anti-neoplastic activity of *T. cruzi* against CRC has been demonstrated in several in vitro and in vivo studies. Rats chronically infected with *T. cruzi* demonstrated a low incidence of chemically induced colon cancer [87]. Additionally, vaccination with *T. cruzi* epimastigote lysates strongly inhibited colon cancer development in a rat model and evoked an integrated anti-tumor response involving both the cellular and humoral components of the immune response. Interestingly, rats developed specific antibodies upon vaccination with *T. cruzi* epimastigote lysates that cross-reacted in vitro with human colon cancer cells. This indicates that *T. cruzi* and colon cancer cells could share common antigens and were capable of mediating antibody-dependent cellular cytotoxicity [52]. A candidate probable antigen is the mucin-type cancer-associated sialyl-Tn antigen (sialic acid-GalNAc-O-Ser/Thr), previously characterized in *T. cruzi* [88]. Thus, protection induced by *T. cruzi* infection or antigens against colon cancer in rat models possibly explains the absence of colorectal cancer in patients with chagasic megacolon [26].

Malaria is a disease caused by an intracellular protozoan parasite, *Plasmodium* (*P.*). It is the most common parasitic infection in both humans and animals. Studies have indicated that *Plasmodium* infection can activate the immune system’s defense against cancer. In a recent experimental study, infecting colon-cancer-bearing mice with *P. yoelii* 17XNL-infected erythrocytes resulted in a reduction in tumor weight and size. This was achieved by inhibiting proliferation and promoting mitochondria-mediated apoptosis in colon cancer cells [89]. It was also reported that the refined malaria protein rVAR2 binds specifically and with high affinity to chondroitin sulfate from various cancer cell lines. This targeted tumor characteristic makes rVAR2 a promising candidate for anti-cancer drug delivery. In fact, a complex formed by conjugating rVAR2 with a hemiasterlin analog (KT886) named (VDC886) was found to have strong cytotoxicity to 33 cancer cell lines in vitro including human colon cancer cells (Colo 205) with inhibitory concentrations 50 (IC50) values ranging from 0.2 pM to 30 nM [90]. Interestingly, in an in vitro study, it was demonstrated that a recombinant circumsporozoite protein, the main surface protein of the malaria parasite’s sporozoite stage, inhibited the proliferation of the human colon cancer cell line, SW480 in a dose-dependent manner. It also induced apoptosis in SW480 cells by suppressing the activation of the nuclear factor κB (NF-κB). Blocking NF-κB activation is a promising strategy for cancer treatment. Further research is needed to investigate the potential use of circumsporozoite protein as a novel NF-κB inhibitor for treating colorectal cancer [91].

*Toxoplasma gondii* (*T. gondii*) is a protozoan parasite that has attracted attention for its potential anti-cancer effects. It infects a wide variety of warm-blooded animals, and about one-third of the world’s population is infected with it. *T. gondii* is able to infect many different types of cells and alter the immune response of the host, which makes it a possible candidate for use in human medicine as a potential oncolytic protozoan [92]. In vitro studies demonstrated that the dense granular protein 16 of *T. gondii* (GRA16) can act as a new telomerase inhibitor, an excellent candidate for gene therapy targeting cancer. It induced apoptosis in HCT116 human colorectal cancer cells by down-regulating the expression of human telomerase reverse transcriptase. This led to the shortening of telomere through the inactivation of telomerase, which was achieved by activating the tumor suppressor phosphatase and tension homolog [93]. Interestingly, conjugation of GRA8, a dense granule protein of *T. gondii*, with the acidity-triggered rational membrane (ATRAM) forming (rATRAM-GRA8-M/AS) induced HCT116 cell death in a dose-dependent manner via mitochondrial metabolic resuscitation pathways. Furthermore, rATRAM-GRA8-M/AS demonstrated significant therapeutic effects in a mouse model with HCT116 xenografts, with noticeable inhibition in tumor growth in mice [94]. Additionally, in a mouse model of colon adenocarcinoma (MC38), intratumoral administration of attenuated *T. gondii* tachyzoites (RH-ΔGRA17) strain with programmed death ligand-1 (PD-L1) blockade significantly improved the survival of mice and halted tumor growth compared to each treatment alone. This treatment also increased lymphocyte infiltration and sensitized the microenvironment in local and distant tumors to systemic PD-L1 blockade treatment [95]. Likewise, intratumoral injection of *Toxoplasma* lysate antigen in CT26 tumor-bearing Athymic nude mice and euthymic mice reduced tumor growth and TIMP-1 level, a metastatic marker, in both mouse models. In Athymic mice, *Toxoplasma* lysate antigen treatment led to a sharp increase in serum IL-12, and MyD88 signals in bone marrow-derived macrophages, suggesting activation of innate immunity. The selective induction of IL-12 by *Toxoplasma* lysate antigen treatment had an anti-tumorigenic effect [96].

In *T. gondii*, the profilin-like protein (TgPLP) acts as a Toll-like receptor (TLR) agonist. A study demonstrated that recombinant profilin-like protein in *T. gondii* (*Tg*PLP) could serve as a TLR-based vaccine adjuvant, enhancing anti-tumor immune responses upon vaccination with autologous whole-tumor cell vaccine (AWV) that was exhibited by prolonged mice survival and smaller CT26 tumors*.* Additionally, *Tg*PLP treatment triggered immune responses as evidenced by increased expression of antigen-presenting cell markers (MHC class I and II, B7.1, and B7.2) in bone marrow-derived macrophage and increased IL-12 and IFN-γ expression in mice. Mice vaccinated with AWV and *Tg*PLP showed more immune cells [CD4^+^ and CD8^+^ T cells, natural killer (NK) cells, and macrophages] in the spleen and higher total IgG and IgG2a concentrations in the blood than mice vaccinated with AWV alone [97].

Similarly, the amino-terminus region of the dense granule protein 6 (GRA6Nt) of *T. gondii* has been shown to activate cancer-specific immunity. In a mouse model of MC38 CRC, the recombinant GRA6Nt (rGRA6Nt) protein acts as an effective adjuvant when combined with non-replicable MC38 colorectal cancer cells treated with mitomycin C or a lethal dose of ionizing radiation. This immunization approach markedly activates protective immunity against replicable MC38 tumor cells challenge, leading to significant inhibition of tumor growth and a marked increase in the infiltration of CD8^+^ T cells in the tumor microenvironment. The CD8^+^ T cells also showed increased activity in secreting granzyme B and IFN-γ in response to cancer cells in vitro. Importantly, this immunization approach is specific to MC38 CRC cells and did not significantly inhibit the growth of EL4 lymphoma tumors [98]. Therefore, both the recombinant Profilin-like protein (r*Tg*PLP) and the recombinant GRA6Nt of *T. gondii* have the potential as powerful adjuvants for cancer cell vaccines used in cancer immunotherapy to effectively activate their specific protective immunity.

Recent studies have shown that the use of an exosome-based strategy instead of live parasite infections could be a safe alternative approach for clinical applications in treating cancer [99, 100]. Exosomes derived from culture supernatants of DCs infected with *T. gondii* Me49 strain induced an anti-tumor effects CRC mouse model. These exosomes significantly slowed tumor growth, inhibited macrophage polarization to M2 phenotype, and regulated the suppressor of cytokine signaling 1 (SOCS1) expression by delivering functional miR-155-5p [100]. Furthermore, a subsequent study showed that these exosomes reduced the proportion of polymorphonuclear granulocytic bone marrow-derived suppressor cells (G-MDSCs, CD11b^+^Ly6G^+^) and monocytic myeloid-derived suppressor cells (M-MDSCs, CD11b^+^Ly6C^+^), while significantly increased the proportion of DCs (CD45^+^CD11c^+^). There was also a significant decrease in the levels of IL-6 and granulocyte–macrophage colony-stimulating factor. The exosomes achieved such an impact by inhibiting the signal transducer and activator of the transcription signaling pathway. Thus, exosomes derived from DCs infected with *T. gondii* could potentially be a new cancer treatment strategy decreasing the proportion of MDSCs and altering the “cold” tumor to a “hot” tumor. These exosomes could also be modified for drug and antibody delivery. These findings could lead to a new approach for treating CRC [99].

*Eimeria* spp*.* is another apicomplexan intestinal protozoan parasite of domestic animals. Interestingly, domestic animals known to maintain this parasite in their small intestinal tissues have a very low incidence of colon cancer. In humans, cyst-forming coccidia parasites belonging to phylum Apicomplexa, with life cycles like *Eimeria*, are maintained through the use of night soil as fertilizer*.* Therefore, a research study investigated the impact of using human excrement (night soil) as a fertilizer on the development of human colon cancer. Japan’s mortality data demonstrated that colon cancer increased after the cessation of night soil use [101]. Thus, the presence of protozoa within intestinal tissue layers may enhance the immune system and prevent colon cancer, and the loss of these organisms could lead to a higher incidence of colon cancer. The authors consider this to be similar to the helminth hypothesis and an extension of the hygiene hypothesis [101].

These findings were further supported by a recent study by Huang and colleagues, who demonstrated that *Eimeria stiedae* (*E. stiedae*) soluble protein is an extremely effective activator of innate immunity in mice. In the murine model of colon cancer (CT26), it enhanced the anti-tumor immune response, inhibited tumor growth, promoted dendritic cell maturation, and activated natural killer cells and CD8^+^ T cells. Additionally, it inhibited angiogenesis and metastasis as demonstrated by the significant reduction in the number of metastatic lung nodules. Thus, *Eimeria* surface protein could play an important role in the treatment of cancer and has the potential to prevent tumor metastasis, which is a leading cause of cancer-related death in patients with colorectal cancer as well as other solid tumors [102].

#### Gastric and esophageal cancers

Gastric and esophageal cancers are prevalent and have high mortality rates. Patients are usually diagnosed at an advanced stage, leading to poor survival outcomes. Despite advancements in cancer treatments, survival rates remain low, highlighting the need for ongoing research to improve treatment strategies [103].

#### Antineoplastic activity of helminth parasites against gastric and esophageal cancers

Studies have shown that the helminth nematode parasites *T. spiralis* and *T. canis* have anti-neoplastic activity against gastric cancer. In an in vitro study, *T. canis* synthesized Troponin protein peptide, a fraction from the excretory–secretory antigens, was able to induce cellular changes and decrease cellular viability in a gastric cancer cell line (AGS) in a dose-dependent manner. It also significantly altered the cellular expression of genes (Mcl-2, APAF1, ZEB1, VEGF, cyclin-D1, and caspase-3) involved in cancer progression [82]. In addition, the extract of *T. spiralis* from adults and newborn larvae inhibited tumor growth in mice grafted with murine forestomach carcinoma (cell line MFC) [104].

#### Antineoplastic activity of protozoan parasites against gastric and esophageal cancers

Apart from helminths, live *T. gondii* tachyzoites have been found to have a significant inhibitory effect on human esophageal carcinoma cells (EC-109 cell line) in a dose-dependent manner [105]. Furthermore, another study demonstrated that the culture supernatant of *T. gondii* inhibited the proliferation and induced apoptosis in human gastric cancer BGC-823 cells in a concentration- and time-dependent manner. This effect may be linked to the up-regulation of p53 expression and the down-regulation of Bcl-2 expression [106]. Interestingly, *T. gondii* infection of a multi-drug-resistant gastric cancer cell line, or its incubation with *T. gondii* cell lysate, has been shown to reduce their resistance to cytostatic drugs by enhancing drug accumulation through reducing the activity of ATP-dependent efflux pump. These findings are intriguing and warrant further investigation [107].

### Hepato-pancreatic cancers

Table 2a, b shows the in vitro and in vivo studies of the anti-neoplastic activity of different parasites against hepato-pancreatic cancers (Hepatocellular Carcinoma and Pancreatic Cancer).

#### Hepatocellular carcinoma (HCC)

Hepatocellular carcinoma (HCC) is a common tumor worldwide and accounts for 85–90% of primary liver cancers. It ranks fifth in frequency and fourth in cancer-related deaths. Loco-regional treatments such as surgical resection, radio-frequency ablation, trans-arterial chemoembolization, or liver transplantation can be effective when HCC is diagnosed early. However, most cases are diagnosed at advanced stages, rendering these treatments ineffective. In such cases, systemic therapy is the only viable option. Unfortunately, neither surgical treatment nor nonsurgical one can effectively improve the outcome of liver cancer patients, highlighting the need for further research to find better treatment options [108].

#### Antineoplastic activity of helminth parasites against HCC

Although infection with *S. haematobium* has been linked to bladder cancer [109], it is less clear and controversial the association between *S. japonicum* infection and hepatocellular carcinoma (HCC). Studies have shown that dysregulated expression of certain miRNAs plays a viable role in the development of various diseases including cancer [110]. Interestingly, research studies concluded that *S. japonicum* miRNA is present in the hepatocytes of mice infected with the parasite, which may enhance the host’s resistance to liver cancer. In fact, in vitro experiments have shown that *Sja*-miRNA-7-5p, *Sja*-miR-3096, *Sja*-miR-61, and *Sja*-miR-71a mimics significantly inhibited the migration of mouse hepatoma (Hepa1-6), human hepatoma (HepG2), and human HCC (SMMC-7721) cell lines [111–114]. Additionally, in a xenograft animal model, mice inoculated with cells transfected with *Sja*-miRNA-7-5p, *Sja*-miR-3096, *Sja*-miR-61, or *Sj*a-miR-71a mimics inhibited tumor growth and experienced significant reductions in tumor volume and weight. This anti-tumor activity was achieved through the modulation of tumor-related genes. For instance, *Sja*-miRNA-7-5p down-regulates the S-phase kinase-associated protein 2 gene [111], *Sja*-miR-3096 targets phosphoinositide 3-kinase class II alpha (PIK3C2 A) gene [112], *Sja*-miR-61 induces an anti-angiogenesis activity by targeting the PGAM1 gene [113], and *Sja*-miR-71a targets the *FZD4* gene [114]. The suppression of tumor growth in vivo by *Sja*-miR suggests that the application of these exogenous miRNAs could potentially provide a novel and promising approach to cancer therapy.

*Fasciola* spp., a liver fluke, is the cause of fascioliasis, a common zoonotic helminth disease. It is a major food-borne disease that currently affects approximately 2 million people in over 70 countries [115]. While two other liver flukes *O. viverrini* and *C. sinensis* have been recognized as definitive causes of cancer [18], fascioliasis caused by *Fasciola hepatica* (*F. hepatica*) has not been linked to cancer. This may be due to the findings of Tsocheva et al*.* [116] who demonstrated that biologically active substances isolated from *F. hepatica* tissue had a significant inhibitory effect on hepatoma cells (MC29 cell line) proliferation.

The nematode helminth parasite *T. spiralis* ensures its survival and immune dialog within the host by releasing excretory–secretory products (ESPs). In vitro studies have shown that a mixture of crude extracts from *T. spiralis* adult worms and newborn larvae exhibited anti-neoplastic activity and induced apoptosis of ascitic hepatoma (cell line H22) and hepatoma cell line (H7402) [104]. Similarly, ESPs from *T. spiralis* adults or muscle larvae inhibited cellular proliferation and induced apoptosis in the liver cancer H22 cell line through the mitochondrial pathway [117]. Furthermore, a *T. spiralis* protein encoded by the A200711 gene was reported to induce apoptosis in human hepatoma (H7402) cells [118]. Recently, *T. spiralis* larval extract (peptide 2 matched the hypothetical protein T01_4238 of *T. spiralis*) induced an in vitro anti-tumor effect on HepG2 cells in a dose-dependent manner, by inducing reactive oxygen species accumulation, leading to the inhibition of cell proliferation [119]. In vivo studies revealed that ICR mice inoculated with mouse ascitic hepatoma H22 cells and C57BL/6 mice inoculated with hepatoma Hepa1-6 carcinoma cells followed by their infection with *T. spiralis* resulted in a significant inhibition in tumor growth [117, 120]. Similar results were obtained by another study where *T. spiralis* infection in hepatocellular carcinoma (HCC) animal model prolonged rats’ survival and decreased progression of the tumor with an increased rate of apoptosis as shown by the decreased expression of Bcl-2 at 30 and 40 days post-infection [121].

*Angiostrongylus cantonensis* (*A. cantonensis*) is another helminth nematode parasite that causes angiostrongyliasis, the most common cause of eosinophilic meningitis in Southeast Asia and the Pacific Basin [122]. *Angiostrongylus cantonensis* ESPs and recombinant Calreticulin (rCRT) (a key effector protein of ESPs) induced apoptosis in human hepatoma HepG2 cells via endoplasmic reticulum stress. Moreover, rCRT had a stronger inhibitory effect on HepG2 cells compared to ESPs while showing no effect on the proliferation of HL-7702 normal human liver cells [123]. In Hepa1-6 mouse tumor models, ESPs from *A. cantonensis* significantly reduced tumor growth [123].

Adult *Setaria equina* (*S. equina*) is a filarial parasite commonly found in the peritoneal cavity of equines worldwide. In a rat model of chemically induced HCC, it was found that repeated administration of *S. equina* ESPs had a beneficial effect on preventing the development of HCC. These products contain antioxidant substances that can reduce oxidative stress by increasing the activity of antioxidant enzymes (CAT, SOD, and GPX), particularly catalase. They also decreased the expression of NF-κB, which plays a role in cancer treatment. Combined treatment with diethylcarbamazine citrate (DEC) was found to modulate the antioxidant effect of *S. equina* ESPs [124]. Another study also showed that repeated doses of this combination therapy (DEC + *Se*ES) had immunomodulatory and protective effects on rat HCC. It increased anti-*S. equina* ESPs IgG, IFN-γ levels, and NK cell infiltration while reducing the expression of MHC I, PCNA, Bcl2, and p53 [125]. Therefore, repeated administration of the combined treatment of DEC and *S. equina* ESPs induced antioxidant, immunomodulatory, and protective effects on rat chemically induced HCC [124, 125].

Interestingly, another study demonstrated the existence of cross-reactive protein bands in *S. equina* adult worms and Huh-7 hepatoma cells at 70 and 75 kDa by anti-DEC and *S. equina* extract antibodies, respectively, using indirect ELISA and western blotting. The existence of shared proteins could also explain the anti-tumor activity of *S. equina* against HCC [126]. Further research is needed to identify and evaluate the antioxidant components of *S. equina* ESPs, as well as the shared proteins with hepatoma cells, as potential novel treatments for HCC.

#### Antineoplastic activity of protozoan parasites against HCC

Infection with *Plasmodium* is known to regulate the host’s immunity by inducing the release of cytokines, activating certain immune cell populations, and altering the function of macrophages [127]. Angsubhakorn and colleagues have shown that *P. berghei* infection in rats reduced the development of hepatic carcinoma induced by dietary aflatoxin B1 [128]. In the same line, another study demonstrated that *P. yoelii 17 XNL* infection effectively inhibited tumor progression in a murine implanted hepatoma model (murine Hepa1–6 or H22 hepatoma cell line) and prolonged their survival time by suppressing tumor angiogenesis. This was attributed to the decreased infiltration of tumor-associated macrophages within the tumor microenvironment that produces matrix metalloprotease 9 (MMP-9) which is well documented to play a crucial role in the process of angiogenesis [129]. Thus, parasite-induced reduction in MMP-9 expression in tumor-associated macrophages resulted in the suppression of tumor angiogenesis. Additionally, *Plasmodium*-derived hemozoin that accumulated in tumor-associated macrophages inhibited IGF-1 signaling through the PI3-K and MAPK signaling pathways and thereby decreased the expression of MMP-9 in tumor-associated macrophages [130].

In another study, Liu et al. [131] validated the use of *P. yoelii* as a novel vaccine vector for hepatocellular carcinoma (HCC) immunotherapy. They reported that *P. yoelii* 17XNL strain expressing murine glypican-3 protein (*P*.*y*-GPC3), which is highly expressed in Hepa1-6 cells, significantly inhibited Hepa1-6-induced tumor growth in the implanted HCC murine model and prolonged their survival time. It also induced a significant GPC3-specific cytotoxic T lymphocyte (CTL) response. This suggests that a *Plasmodium*-based vector is highly effective in inducing tumor antigen-specific T-cell-mediated immunity. Additionally, it increased concentrations of Th1-associated cytokines, such as IL-2, IFN-γ, and TNF-α, in serum and favored expansion of the CD8α^+^ dendritic cell subset with higher expression of CD80 and CD86 molecules [131]. Moreover, *P. yoelii* infection alone inhibited the recurrence and metastasis of HCC and improved the prognosis in both non‑resection and resection murine orthotopic HCC models. This was achieved through suppressing CC‑chemokine receptor 10‑mediated PI3K/Akt/GSK‑3β/Snail signaling and preventing epithelial–mesenchymal transition. These findings show potential for the development of new therapies for HCC recurrence and metastasis [132]. As a result, a clinical trial of *Plasmodium* immunotherapy for advanced liver and breast cancer [NCT03474822] has been approved and is currently underway in China [36].

A study on the apicomplexan protozoan parasite *T. gondii* has shown that *T. gondii* tachyzoites RH strain inhibited the in vitro proliferation and induced apoptosis of HCC (H7402) in a concentration-dependent manner. This effect was associated with the down-regulation of *cyclinB1* and *cdc2* (cell cycle-related genes), an increase in the apoptosis-related protein Caspase-3, and a decrease in Bcl-2 expression [133].

Another study demonstrated that GRA16, a dense granule protein of *T. gondii*, has anti-cancer activity against hepatocellular carcinoma. In an in vitro study, GRA16 decreased cell proliferation, anti-apoptotic factors, p‐AKT/AKT ratio, cell migration, and invasive activity of hepatocellular carcinoma (HepG2) cells. In addition, it decreased tumor size in HepG2 cell‐xenograft nude mice. The study revealed that GRA16 acts as a *Herpes virus*‐associated ubiquitin‐specific protease inhibitor, targeting the nuclear localization of phosphatase and tensin homolog and inducing an anti-cancer effect against HCC in a p53‐dependent manner [134]. Similarly, the effector molecule *Toxo*GRA15II; a *T. gondii* virulence-associated molecule of dense granule protein, can induce macrophage polarization to M1. An in vitro study demonstrated that *Toxo*GRA15II-polarized macrophage induced significant inhibition of migration and invasion of the Hepa1-6 cells and decreased expressions of matrix metalloproteinase (MMP)-9 and MMP-2 without notable apoptosis. In parallel to that study, *Toxo*GRA15II-polarized macrophages inoculated to tumor-bearing C57BL/6 mice induced marked restriction of tumor growth through its related cytokines profile in tumor and spleen tissues. Thus, *Toxo*GRA15II, due to its ability to activate specific cell populations early while being non-toxic to mammals, could be a promising new approach to enhance host innate immunity to fight cancer [135].

#### Pancreatic cancer [pancreatic ductal adenocarcinoma (PDAC)]

Pancreatic ductal adenocarcinoma (PDAC) is a lethal disease and is expected to become the second leading cause of cancer death in USA by 2030 [136]. Yet, multiple challenges are obvious in its treatment including limitations in patients who are eligible for surgical tumor resection, and a high rate of tumor recurrence (around 70%). Additionally, immune checkpoint inhibitors are not effective in controlling most PDAC tumors due to the tumor’s strong immunosuppressive microenvironment and lack of immune cell infiltration [137]. Therefore, it is crucial to find new methods to improve the effectiveness of immunotherapy in PDAC.

#### Antineoplastic activity of helminth parasites against PDAC

Recently, *E. granulosus* has been studied concerning pancreatic cancer. Induction of secondary hydatidosis in a rat model of pancreatic cancer induced by azaserine significantly inhibited the development of precursor foci of neoplastic changes in the pancreas. Although the study used live protoscolex to induce secondary hydatidosis, the authors highlighted that using dead protoscolex yielded the same results in their pilot study [138]. Thus, exploring the antigenic derivatives of this parasite against pancreatic cancer is noteworthy to be investigated.

#### Antineoplastic activity of protozoan parasites against PDAC

There is evidence suggesting that certain protozoan parasites, such as *T. gondii*, have the potential to be used as an effective immunotherapeutic agent even in an immunosuppressive cancer such as PDAC. Thus, a number of studies investigated that theory with attenuated strains of *T. gondii* gaining the biggest chunk of those trials. Attenuated auxotroph strain of virulent *T. gondii* (cps), which is capable of selective invasion of cells without replication, promoted immunotherapeutic effect against PDAC [139–141]. Treating pancreatic ductal adenocarcinoma in mice by intratumoral inoculation of the attenuated *T. gondii* strain cps induced regression of established tumors in mice [139]. In another study, the treatment of established aggressive disseminated pancreatic cancer in a mouse model with the attenuated *T. gondii* strain cps induced stimulation of the immune system at a multi-level platform. It promoted a significant decrease in tumor-associated macrophages, a marked increase in DCs infiltration of the pancreatic tumor microenvironment, an increase in the expression of co-stimulatory molecules CD80 and CD86, and the production of IL-12. Additionally, cps treatment led to an increase in CD4^+^ and CD8^+^ T-cell infiltration into the tumor microenvironment, activation of tumor-resident T cells, and increased production of IFN-γ [140]. Furthermore, mice that survived the primary pancreatic tumor after cps treatment showed strong immune protection against the recurrence of pancreatic cancer. These findings indicate that a variety of cell-mediated and humoral antibody-mediated anti-tumor immune responses generated by primary immunotherapy may be crucial in developing long-lasting protective immunity against aggressive cancers like PDAC [141]. These studies highlight the importance of targeting and neutralizing suppressive myeloid cell populations as an effective immunotherapeutic mechanism to fight pancreatic cancer. They also identified cps immunotherapy as the first treatment to provide a long-term survival benefit [140, 141].

In another study, the use of another variant of attenuated *Toxoplasma* (NRTUA strain) as a monotherapy inhibited PDAC tumor growth in a subcutaneous mouse model by activation of DCs and increasing CD8^+^ T-cell infiltration in the tumor microenvironment. NRTUA strain efficiently enhanced the anti-tumor activity of anti-PD1 antibody when used in combination, induced a significant immune response, and controlled tumor growth in Pan02 tumor-bearing mice. This was achieved by targeting the tumor immunosuppressive myeloid-derived suppressor cell population and modifying the tumor microenvironment [142].

For safety concerns, it is worth noting that instead of using live parasites, Pyan and colleagues demonstrated that treatment with soluble *T. gondii* or *T. gondii* profilin in pancreatic cancer tumors in C57BL/6J mice decreased tumor volume and increased tumor infiltration with CD4^+^ and CD8^+^ T cells [98, 143]. These studies suggest that *T. gondii* and its derived products could be promising targets for novel pancreatic cancer treatment, which could potentially be translated into future therapeutics for this most aggressive, resistant, and lethal cancer.

### Lung cancer

Table 3 shows the in vitro and in vivo studies of the anti-neoplastic activity of different parasites against lung cancer.

Lung cancer is a highly common and deadly form of cancer, causing approximately 1.35 million deaths each year and accounting for around 30% of all cancer-related deaths worldwide [144]. There are two main types of lung cancer: small cell (SCLC) and non-small cell lung cancer (NSCLC). The latter accounts for over 80% of lung cancer cases [1]. The development of drug resistance and frequency of metastasis are important factors imposing its high mortality rate. Even with aggressive treatments like surgery, radiation, and chemotherapy, the survival rates are still low [145, 146]. Contrarily, biological therapy is considered a safe and effective treatment approach, justifying the conduction of numerous experimental studies including those investigating the anti-neoplastic activity of parasites.

#### Antineoplastic activity of helminth parasites against lung cancer

Several studies have shown the anti-tumor activity of *E. granulosus* against lung cancer. In a murine model, vaccination with human hydatid cyst fluid induced a strong anti-tumor immune response involving both the innate and adaptive immune components that significantly inhibited Lewis lung carcinoma (LLC) cell growth both in prophylactic and therapeutic settings and significantly increased mouse survival. Flow cytometry analysis revealed that hydatid cyst fluid-specific antibodies recognized both membrane and intracellular molecules in lung cancer cells [147]. This is in agreement with the earlier result obtained by immunoelectrophoretic analysis where a broad and intense band was formed when the serum from a patient suffering from pulmonary carcinoma was tested using hydatid cyst fluid [148]. It also supports the potential of anti-hydatid cyst fluid antibodies to generate an anti-tumor effect, as observed in vitro with the serum of patients with hydatid cyst and their ability to induce a cytotoxic effect on NCI-H209/An1 human SCLC cells [149].

The anti-tumor effect of *T. spiralis* was observed against lung cancer cells (A549) inoculated into a *T. spiralis*-infected animal which was dependent on the time of tumor challenge to *T. spiralis* infection [150]. In other studies, the inhibition of proliferation and induction of apoptosis was observed in SCLC cells (H446 SCLC) and the NSCLC cells (A549) when co-cultured with *T. spiralis* muscle larvae excretory–secretory proteins. This effect was found to be dose and time-dependent. The apoptosis was triggered by activating the mitochondrial apoptotic pathway, as evidenced by the low expression of anti-apoptotic genes (Bcl-2 and Livin) and increased expression of pro-apoptosis genes (sit-in, Apaf-1, caspase-9, and caspase-3) [151, 152]. Furthermore, *T. spiralis* muscle larvae proteins significantly altered the transcriptome profiling of A549 lung cancer cells, with the expression of 2860 genes being altered. There were an up-regulation of 1634 genes and a down-regulation of 1226 genes, which are known to be associated with pathways in cancer and metabolic processes. Moreover, there was a down-regulation of glycolysis-related genes which may be detrimental to A549 cells [153]. A small Heat Shock Protein (sHSP-DQ 986457) was identified as an associated antigen between *T. spiralis* and LLC cells, and Western blotting analysis revealed that recombinant sHSP reacted with anti-LLC cell antiserum [154]. *Trichinella spiralis* sHSP using invasive *Lactobacillus plantarum* (NC8) as a vector vaccine (NC8-sHSP) successfully inhibited tumor growth in both prophylactic and therapeutic experiments in the LLC murine model, through the induction of humoral, cellular, and mucosal immunity [155].

#### Antineoplastic activity of protozoan parasites against lung cancer

Several studies have shown that certain protozoal infections, such as *T. cruzi*, *Trichomonas vaginalis* (*T. vaginalis*), *Plasmodium* spp., and *T. gondii*, and their derivatives have been deeply investigated experimentally and have successfully shown anti-neoplastic activity against lung cancer and can interfere with tumor growth [156–160].

*Trichomonas vaginalis* is a flagellated protozoan parasite that causes trichomoniasis, a sexually transmitted disease [161]*.* It typically inhabits the genitourinary tract, but there have been cases, supported by molecular identification, where it has been found in the respiratory tract of neonates born to mothers infected with *T. vaginalis* and in adults with a history of urogenital contact [162]. In an in vitro study, it was found that *T. vaginalis* can adhere, cause cytotoxic effects, and induce apoptosis in the human lung alveolar basal carcinoma epithelial cell line A549 [158].

In a pre-clinical murine lung cancer model, an anti-tumoral effect was achieved by a protein lysate derived from *T. cruzi* epimastigote both in preventive and therapeutic settings. It prevented tumor growth and induced both humoral and cellular anti-tumor immune responses. This was associated with the activation of natural killer cells, INF-γ production, and tumor cell cytotoxicity. Flow cytometry analysis showed that the antibodies induced by *T. cruz*i lysate recognized both membrane and intracellular molecules in lung cancer cells. The anti-tumor effects of *T. cruzi* lysate are likely due to its ability to activate both innate and adaptive immune responses, with carbohydrate components playing a crucial role [157].

In addition to their immunostimulatory role in cancer therapy, parasites can also contribute to cancer gene therapy through certain enzymes. One such enzyme is hypoxanthine–guanine phosphoribosyltransferase (HGPRT), which is produced by the parasite *Trypanosoma brucei* (*T. brucei*), the cause of African sleeping sickness disease. Hypoxanthine–guanine phosphoribosyltransferase can convert allopurinol, a purine analog, into nucleotides more effectively than its human homologue. A study generated a new suicide gene/prodrug system showed that specific cancer cells modified to express *Tb*HGPRT were able to convert the non-toxic allopurinol into cytotoxic metabolites, selectively induced apoptosis in five NSCLC cell lines, one murine lung cancer cell line (M27), and four human NSCLC cell lines (A549, H460, H661, H520). However, the bronchioalveolar carcinoma cell line (H322), mouse mammary tumor cell line (DA3), three human breast carcinoma cell lines (MCF7, MDA231, ZR75), and human hepatocellular carcinoma (HepG2) and neuroblastoma (IMR32) cell lines were not affected by the treatment. The reason for the selective efficacy in certain cancer cell lines is unknown. Neither the expression of the *Tb*HGPRT gene nor the cellular accumulation of the prodrug are responsible for the differences in cytotoxicity observed among the cell lines tested. However, this differential selectivity is an important observation and can be used to improve prodrug-based suicide gene therapy. Other allopurinol derivatives or parasitic enzymes can be explored for therapeutic use. The development of the *Tb*HGPRT/allopurinol system has the potential to be a therapeutic approach for gene therapy of lung cancer [163].

Malaria has been found to stimulate the immune system in the host, which is believed to be effective in fighting certain types of cancer [36]. In a murine LLC model, *Plasmodium* infection inhibited the growth and metastasis of lung cancer and increased the survival of tumor-bearing mice. Malaria infection induced anti-tumoral effects via triggering a strong immune response, releasing IFN-γ and TNF-α, and the activation of NK cells. It also enhanced the body’s adaptive immune response, leading to increased proliferation of tumor-specific T cells and the activity of CD8^+^ T cells, as well as inhibiting angiogenesis. Additionally, when combined with a lung cancer DNA vaccine [pcDNA3.1-hMUC1], malaria infection boosted the immune response and had a synergistic effect against the tumor [164]. Within the same context, *Plasmodium* infection inhibited the accumulation of immunosuppressive myeloid-derived suppressor cells and Tregs with a consequent elevation of CD8^+^ T-cell-mediated cytotoxicity within the tumor microenvironment. Furthermore, it inhibited the release of cytokines and chemokines from tumors by releasing exosome-like vesicles rather than direct cell-to-cell contact [165]. Consequently, intratumoral injection of plasma exosomes derived from *Plasmodium*-infected mice significantly reduced the growth of LLC in mice. This was achieved by inhibiting tumor angiogenesis through plasma exosomes containing endogenous functional microRNAs [160].

Parallel to those studies, immunization with genetically attenuated *Plasmodium* sporozoites activated innate immunity and inhibited lung growth and angiogenesis [166]. Interestingly, when these attenuated *Plasmodium* sporozoites were used as a vector for a lung cancer-specific antigen (MAGE-A3), they induced strong MAGE-A3-specific CD8^+^ T-cell responses, inhibited tumor growth, and increased the survival rate of mice [167].

Interestingly, *Plasmodium* circumsporozoite protein significantly inhibited autophagy, making A549 and H1975 lung cancer cell lines more sensitive to Gefitinib showing a significant increase in apoptosis and decreased cell viability. Moreover, in nude mice with lung adenocarcinoma cells xenografts, inhibition of autophagy by *Plasmodium* circumsporozoite protein suppressed cell growth and increased apoptosis when exposed to Gefitinib. Therefore, *Plasmodium* circumsporozoite protein could potentially be used as an autophagy inhibitor to enhance the effectiveness of tyrosine kinase inhibitors target therapy for epidermal growth factor receptor-mutant-resistant lung adenocarcinoma [168].

In the same line, another study found that recombinant circumsporozoite protein has a dose-dependent inhibitory effect on the proliferation of the human lung cancer cell line A549. It also triggered apoptosis by suppressing the activation of the NF-κB [169]. These findings are similar to the previous research results on the human colon cancer cell line SW480 [91].

Infection of LLC-bearing mice with the non-lethal *P. yoelii* was found to inhibit tumor growth. This was achieved via up-regulation of F63, a novel long non-coding RNA (lncRNA) that inhibited the secretion of tumor vascular endothelial growth factor A and endothelial cell clone formation, migration, invasion, and tube formation. The study suggested that F63 could be a promising candidate for gene therapy in lung cancer [170]. Moreover, combined therapy of *Plasmodium* immunotherapy (*P. yoelii* infection) with radiotherapy significantly inhibited tumor growth and extended the life span of tumor-bearing mice in the subcutaneous model of mouse NSLLC. The combination therapy was more effective in up-regulating the perforin-expressing effector CD8^+^ T cells and down-regulating the myeloid-derived suppressor cells, thus is considered effective cancer treatment [171].

*Plasmodium chabaudi*, a rodent *Plasmodium* spp., is extensively used to model human infection in laboratory mice. Infection of mice with *P. chabaudi* in a subcutaneously implanted murine LLC model, either alone or in combination with Gemcitabine treatment significantly inhibited tumor growth and metastasis. The combination therapy was more effective than the monotherapy with significant prolongation of mice survival, and evidence of a synergistic inhibitory effect. This may be partially attributed to the inhibition of the epithelial–mesenchymal transition of tumor cells, because of blocking the CXCR2/TGF-β-mediated PI3K/Akt/GSK-3β/Snail signaling pathway. Hence, it is noteworthy to consider introducing *Plasmodium* immunotherapy and gemcitabine combination to clinical trials for lung cancer patients [172].

Based on the ability of *Plasmodium* to induce a strong anti-tumor effect, clinical trials (Phase 1–2) of *Plasmodium* immunotherapy for advanced NSCLC (ClinicalTrials.gov ID NCT02786589) have been approved and are ongoing in China [36]. The clinical trial for advanced NSCLC began in 2016 and was originally supposed to be completed in 2020. However, the estimated study completion has been extended to December 2023, most likely due to the negative impact of the *Corona* virus epidemic. These trials aim to improve the outcomes for advanced NSCLC patients. Controlled infection with blood-stage *Plasmodium vivax* by co-administration of antimalarial drugs could obtain a safe level of parasitemia capable of activating the immune response without complications [173]*.* Thus, it is expected that genetically modified attenuated human malaria parasites could offer a significant opportunity to be explored as a potential cancer vaccine vector or as a form of immunotherapy for cancer patients.

Regarding the protozoan parasite *T. gondii*, in vitro study has shown that live *T. gondii* tachyzoites have a significant inhibitory effect and induced cell apoptosis in non-small-cell lung cancer cells (cell line A549) in a dose-dependent manner with up-regulation of p53 and down-regulation of Bcl-2 gene expression. Numerous in vivo studies have demonstrated the anti-cancer activity of *T. gondii* against lung cancer. Intralesional injection of formalin-fixed *Toxoplasma* tachyzoites induced potent anti-tumor effects against LLC [159]. Furthermore, infection of mice with *T. gondii M49* strain inhibited LLC growth in the murine model by inducing Th1 immune responses and inhibiting angiogenesis [174]. Moreover, *T. gondii* excretory–secretory antigens inhibited tumor growth in LLC-bearing mice and reduced the proportion of splenic Treg cells in splenocytes [175]. Interestingly, the dense granule protein of *T. gondii* (GRA16) demonstrated anti-cancer activity by reducing tumor size in a mouse xenograft model of NSCLC. Interestingly, it enhanced the effects of the anti-cancer drug Irinotecan. The co-administration of Irinotecan and GRA16 promoted the anti-cancer effects of Irinotecan by inhibiting NF-κB and cell cycle arrest induced by GRA16, ultimately increasing the effectiveness of Irinotecan in treating non-small-cell lung carcinoma [176]. Furthermore, a combination therapy using intratumoral administration of attenuated RH-ΔGRA17 *T. gondii* strain tachyzoites, along with programmed death ligand-1 (PD-L1) blockade, induced synergistic therapeutic activity. This resulted in the up-regulation of innate and adaptive immune responses, increased lymphocyte infiltration, and sensitized the microenvironment in local and distant tumors to systemic PD-L1 blockade treatment. This ultimately led to a reduction in tumor size and improved survival in most treated mice [95].

### Breast cancer

Table 4 shows the in vitro and in vivo studies of the anti-neoplastic activity of different parasites against breast cancer.

Breast cancer is the most prevalent and heterogeneous malignancy affecting women worldwide. It is also a leading cause of cancer-related deaths in women. The main treatments include surgery, radiation, hormonal therapy, and chemotherapy. However, chemotherapy has adverse effects due to being non-selective in targeting cancer cells. Furthermore, resistance to therapy is a major emerging challenge. Therefore, it is crucial to discover more effective anti-cancer treatments with fewer side effects [177, 178].

#### Antineoplastic activity of helminth parasites against breast cancer

Several studies have shown that hydatid cyst fluid has anti-tumor activity against breast cancer. In vitro study revealed that hydatid cyst fluid antigens, such as antigen B, glycolipid, glycoprotein, and 78 kDa fraction-induced apoptosis in mouse breast cancer cells (4T1 cell line) with the highest apoptotic percentage observed with 78 kDa fraction and glycoprotein fractions [179]. An in vivo study demonstrated that intraperitoneal hydatidosis in a rat model of Dimethyl Benzanthracene (DMBA) breast cancer reduced the development of breast cancer tumors by 50% [180]. In other studies, prophylactic immunization with the hydatid cyst wall protein bands (~ 27/28 kDa), that showed cross-reactivity with breast cancer patients’ sera, or crude hydatid cyst wall antigens (laminated layer) caused significant inhibition of 4T1 breast tumor growth, decrease of metastasis, and prolongation of the tumor-bearing mice survival time through a rise in the levels of IL-2, TNF-α, IFN-γ, and IL-4 [51, 181]. Mass spectrometry analysis revealed that this ~ 27/28 kDa band has several proteins/polypeptides with a high degree of homology with cancer cell antigens, which are responsible for the cross-reactivity and anti-tumor immune response [51]. In the same line, similar findings were reported following immunization with hydatid cyst fluid and antigen B on 4T1 breast tumor cells in BALB/c mice. The anti-tumor activity of hydatid cyst fluid and antigen B may be related to the immune responses against these antigens with polarization of the Th1/Th2 ratio toward the Th1 pathway [182].

Parasites produce a variety of protease inhibitors with different functions primarily to evade their host [183]. For example, *E*. *granulosus* KI-1 (EgKI-1), a member of the Kunitz-type protease inhibitor family, is highly expressed by the oncosphere of *E. granulosus*. It is a powerful inhibitor of neutrophil elastase and chemotaxis [184] and was recently granted an international Patent Publication [185]. A study showed that EgKI-1 can penetrate tumors and effectively interact with and kill tumor cells. In vitro study demonstrated that recombinant EgKI-1 inhibited the growth and migration of breast cancer cells (MCF-7, T47D, and MDA-MB-231 cell lines) in a dose-dependent manner by disrupting the cell cycle and inducing apoptosis. Moreover, in a mouse model of triple-negative breast cancer (MDA-MB-231), rEgKI-1 treatment led to a significant reduction in tumor growth. This suggests that EgKI-1 has the potential to be a promising molecule for the development of anti-cancer therapeutics, as protease inhibitors are important in the treatment of cancer due to their association with carcinogenesis and cancer progression [186].

The anti-tumor activity of *E. granulosus* against breast cancer could be related to the mutual cross-reaction that exists between antigens of hydatid cyst and cancer cells. In vitro study demonstrated that hydatid cyst fluid antigens (Antigen B, glycolipid, glycoprotein, 78 kDa fractions cyst antigens, and excretory–secretory antigen of protoscolices) in particular the 78 kDa and glycoprotein fractions induced apoptosis on breast cancer cells (4T1 cell line) [179]. Additionally, rabbit antisera raised against hydatid cyst antigens (hydatid cyst fluid, germinal and laminated, protoscolex, and excretory–secretory) induced cell death of 4T1 breast cancer cell line [187]. Moreover, it has been shown using flow cytometry that rabbit antisera raised against hydatid cyst antigens (hydatid cyst wall, protoscolex, and cystic fluid antigens) reacted with breast cancer cells (4T1) [51, 188]. Western immunoblotting demonstrated a considerable cross-reactivity between hydatid cyst wall antigens and breast cancer patients’ sera at a specific band (~ 27/28 kDa) [51]. Also, antisera raised against laminated and germinal layers of hydatid cyst reacted with excretory–secretory products of breast cancer cells. Moreover, a reaction was detected between hydatid cyst antigens (hydatid fluid, laminated and germinal layer antigens, and excretory–secretory antigens of protoscolices) and sera of patients with breast cancers [48].

Another study also observed cross-reactivity in *S. equina* parasite and MCF-7 human breast cancer using rabbit anti-*S. equina* extract and anti-diethylcarbamazine citrate polyclonal IgG antibodies, using indirect ELISA and western blotting. The results revealed the presence of cross-reactive proteins at molecular weights of 75 kDa and 70 kDa, which may correspond to G6PD and HSP70, respectively [89, 126]. Further research is required to characterize, purify, and evaluate the efficacy of these shared proteins as potential vaccine candidates for breast cancer treatment.

In another in vitro study, recombinant *T. solium* calreticulin (rTsCRT) either alone or in combination with 5-fluorouracil inhibited the growth of the breast cancer cell line (MCF7) by interacting with scavenger receptors [75].

*Trichinella spiralis* parasite shifts the host immune response from a Th1 profile, characterized by pro-inflammatory cytokines, to a Th2 profile with the release of different cytokines with anti-inflammatory properties. Interestingly, *T. spiralis* infection successfully inhibited tumor growth and prolonged host survival. In 1970, Weatherly discovered the prolongation of survival rates and reduction of mortality of Swiss mice model of breast tumor when sublethally infected with *T. spiralis* muscle larvae [189]. Similar results were also demonstrated by Apanasevich and Britov who reported that *T. spiralis* infection inhibited the growth of chemically induced mammary gland cancer by 7,12-dimethylbenzo(a)anthracene [190].

#### Antineoplastic activity of protozoan parasites against breast cancer

Live attenuated parasites allow the host’s immune system to interact with a wide range of antigens that are important for the development of protective immunity without causing harm. In this context, a study by Caner et al. [191] demonstrated that intratumoral inoculation of attenuated *Leishmania* strains (*L. infantum* and *L. major*) regressed 4T1 breast cancer in BALB/c. This also led to the activation of an anti-tumor immune response, inducing M1 phenotype-macrophages and subsequently triggering the adaptive immune response [191].

*Trypanosoma cruzi* epimastigotes lysate showed a direct inhibitory effect on in vitro MCF-7 cells human breast cancer cells [192]. Studies in animal models have demonstrated that vaccination with *T. cruzi* epimastigote lysates strongly inhibited chemically induced mammary carcinoma. This vaccination-induced activation of both CD4^+^ and CD8^+^ T cells and higher splenocyte cytotoxic responses against tumors. This was associated with an increase in the number of CD11b/c^+^ His48^−^ MHC II^+^ cells corresponding to macrophages and/or DCs, which exhibited augmented NADPH-oxidase activity. Additionally, antibodies specific for mammary rat cancer cells were capable of mediating antibody-dependent cellular cytotoxicity. This anti-cancer effect was due to an integrated immune response involving both the cellular and humoral components. In the same study, anti-*T. cruzi* antibodies were cross-reacted with breast cancer cell lines, denoting the existence of shared epitopes between *T. cruzi* and breast cancer cells [52]. In other studies, *T. cruzi* calreticulin (rTcCRT) was documented to have anti-angiogenic and anti-tumor properties, inhibiting the growth of the murine mammary (TA3 MTXR tumor cell line) in vivo [193, 194]. Similarly, a study demonstrated that *T. cruzi* infection and *T. cruzi* epimastigotes extract inhibited the growth of an aggressive murine mammary adenocarcinoma cell line (TA3-MTXR) in mice. That study concluded that translocated/externalized natural *T. cruzi* Calreticulin (nTcCRT) played a significant role in the anti-mammary tumor effect during experimental *T. cruzi* infection [195]. An in vitro study demonstrated that treating a murine mammary adenocarcinoma cells (TA3 cell line) with rTcCalr enhances phagocytosis and alters the expression of a variety of membrane molecules. This includes decreasing the membrane expression of MHC class II, m-Dectin-1, Galectin-9 and PD-L1, while increasing the expression of Rae-1γ, which is associated with increased tumor immunogenicity [196].

Interestingly, both the native and recombinant forms of *T. cruzi* calreticulin showed an anti-mammary tumor capacity in vivo. They were also found to be more effective than human calreticulin in their anti-angiogenic effect as well as in inhibiting endothelial cell migration and differentiation, thus suppressing tumor growth [194, 195].

*Trypanosoma cruzi* P21 is a protein secreted by the parasite and its recombinant form rP21 has various biological properties. These include binding to CXCR4 receptors in macrophages, chemotactic activity of immune cells, and inhibiting angiogenesis [197, 198]. A study reported the binding of *T. cruzi* rP21 to triple-negative breast cancer (MDA-MB-231 cells) cell membrane’s CXCR4 receptors and down-regulating CXCR4 gene expression. This was associated with a decrease in receptors in the cytoplasm of MDA-MB-231 cells, indicating CXCR4 internalization. This internalization probably explains the desensitization of the receptors in these cells. As a result, rP21 was found to reduce migration, invasion, and progression in MDA-MB-231 cells [197].

In another study, *T. cruz*i infection showed a high invasion and multiplication rate in the triple-negative breast cancer cell line (MDA-MB-231). This infection contributed to the perpetuation of a cycle of MDA-MB-231 cell proliferation, parasite cell invasion, infected cell lysis, and impaired host cell migration. These findings suggest that molecules from *T. cruzi* may hinder host cell migration with potential use to avoid metastasis [199].

The recombinant malarial protein VAR2–hemiasterlin Drug (KT886) Conjugate (VDC886) has shown strong anti-cancer activity in both in vitro and in vivo studies. In in vitro testing, results showed a strong cytotoxic effect on human breast cancer cells (MDA-MB-231 and 4T1 breast cancer cell lines). Moreover, in vivo testing in a highly aggressive syngeneic mouse model of bone metastatic murine breast cancer (4T1), administration of VDC886 resulted in a significant increase in mice survival time with no detectable metastases compared to control mice, all of which died with metastatic disease [90]. Also, infection with the non-lethal *P. yoelii 17XNL* strain significantly inhibited tumor growth, and increased the survival rate of mice with triple-negative breast cancer (4T1). These effects might be due to the induction of CD8^+^ T-cell-mediated anti-tumor immune responses [200]. A clinical trial of *Plasmodium* immunotherapy for advanced breast cancer [NCT03474822] has been approved and is currently ongoing in China [36].

Several studies have shown that *T. gondii* and its antibodies have an inhibitory effect on the growth of breast cancer [187, 201, 202]. In vitro studies, live *T. gondii* tachyzoites had a significant dose-dependent inhibitory and cytotoxic effect on MCF-7 cells, a human breast cancer cell line [105, 201]. Another study showed that these tachyzoites were able to effectively destroy Her2/Neu-expressing mammary cancer cell lines (TUBO cells) in a dose- and time-dependent manner [202]. Moreover, rabbit anti-*T. gondii* antisera also induced significant death of the 4T1 breast cancer cell line [187]. Additionally, it has been shown by flow cytometry that both rabbit and human anti-*T. gondii* antisera reacted strongly with breast cancer cells (4T1 and MCF7) [53, 203], suggesting the existence of shared epitopes on the surface of cancer cells and *Toxoplasma* antigens. These findings highlight the potential use of these antibodies in targeted cancer immunotherapy. Similarly, *T. gondii* anti-cancer activity was reported in several animal studies. Chronic *T. gondii* infection led to significant resistance to mammary tumor development in C_3_H/_He_ mice [204]. Moreover, intratumoral inoculation of an attenuated uracil auxotroph strain of *T. gondii* directly into the 4T1 tumor stimulated anti-tumor immunity in mice, resulting in inhibition of tumor growth and metastasis, increased survival of the tumor-bearing mice, and increased secretion of IL-12 and IFN-γ in both the serum and tumor microenvironment [205]. In agreement with the previous results from animal studies, the protective effect of *T. gondii* infection in breast cancer patients was also reported. In a hospital-based cohort study, Ye et al. [206] demonstrated a favorable effect of high anti-*T. gondii* IgG on breast cancer survival, particularly in patients with systemic inflammation and high levels of IL-17 or IL-9. These results support the potential use of attenuated *T. gondii* in breast cancer immunotherapy [206].

*Neospora caninum* (*N. caninum*), an intracellular protozoan parasite, similar to *T. gondii* and is a major cause of reproductive failure in dairy cattle worldwide. In humans, it has not been shown to cause zoonotic disease, making it a potentially safe microbial anti-cancer therapy [207]. In an in vitro study, *N. caninum* was found to invade and multiply within human breast carcinoma cells (MCF-7) and destroy them [208].

*Besnoitia jellisoni* (*B. jellisoni*) is a rodent intracellular protozoan parasite closely related to other tissue-cyst-forming apicomplexan parasites such as *T. gondii* and *N. caninum* [209]*.* Similar to *T. gondii* infection, chronic infection with *B. jellisoni* induced significant resistance to mammary tumor development in C_3_H/_He_ mice [204].

### Hematological malignancies

Table 5a–d shows the in vitro and in vivo studies of the anti-neoplastic activity of different parasites against hematological malignancies (Leukemia, Lymphomas, Multiple Myeloma, and Mastocytoma).

#### Leukemia

Leukemia is a group of malignant hematologic disorders. The incidence of the disease is approximately 15 cases per 100,000 individuals. Current treatments for leukemia are not satisfactory, so new therapeutic strategies need to be developed [210].

#### Antineoplastic activity of helminth parasites against leukemia

Certain helminth parasites have been found to have anti-leukemia activity. Studies have shown that the recombinant *Sj*16 (r*Sj*16), a secreted protein from *S. japonicum*, has been found to significantly suppress the proliferation of murine myelomonocytic leukemia cell line (WEHI-3B JCS) cells in a dose- and time-dependent manner by inducing G1/G0 cell cycle arrest and apoptosis. This is achieved through the activation of the caspase-mediated mechanism by regulating the expression of the Bcl-2 family [38, 211].

The protoscolex hydatid cyst somatic antigen showed a similar anti-leukemic effect in vitro by inducing programmed cell death in K562 cells (a human chronic myeloid leukemia cell line) in a dose and time-dependent manner [212].

Similarly, in other in vitro studies, crude *T. spiralis* antigen and *T. gondii* tachyzoites inhibited the proliferation and induced K562 cell apoptosis [104, 213]. Interestingly, another study revealed that the excretory/secretory products from muscle larvae *T. spiralis* induced a dose-dependent proliferative response in U937 cells, an acute myeloid leukemia cell line, at 24 h and 48 h of treatment. However, after 72 h, significant cell death was observed, with a significantly higher apoptotic index than untreated cells [214]. In addition, peritoneal macrophages from *T. spiralis*-infected mice 1 week post-infection showed a strong cytotoxic effect on mouse R1 leukemia cells in vitro [215]*.*

The anti-leukemia activity was also demonstrated with the somatic antigen of *Marshallagia marshalli* (*M. marshalli*), a common gastrointestinal nematode worm that infects sheep. It inhibited proliferation and induced apoptosis in human chronic myeloid leukemia K562 cells [216].

#### Antineoplastic activity of protozoan parasites against leukemia

Interestingly, Eligio García et al. [217] demonstrated that the antigenic proteins of *T. cruzi* are shared with acute lymphoblastic leukemia. This was demonstrated by the reactivity of rabbit polyclonal antibodies against *T. cruzi* with protein extracts from acute lymphoblastic leukemia cells. This finding suggests an anti-parasitic immune response that also cross-reacts with cancer cells. Therefore, it is important to identify the antigens involved and determine their potential as target cells in cancer therapy [217].

As regards *Plasmodium*, a research study showed that VDC886, a hemiasterlin analog (KT886) conjugated to recombinant malaria protein VAR2 (rVAR2), as a drug carrier induced a strong in vitro cytotoxicity to myeloid leukemia cells (KG-1 cell line) [90].

In another study, infection of mice with the malaria parasite, *P. yoelii* significantly reduced the growth of WEHI-3 cells in mice. Additionally, there was a marked decrease in tumor cell infiltration in the liver and spleen. *Plasmodium* inhibited murine leukemia by boosting immune responses. It increased CD3 T-cell surface marker and CD19 B-cell surface marker while decreasing the surface markers of monocytes (CD11b) and macrophages (Mac-3). Additionally, it stimulated the secretion of IFN-γ and TNF-α and enhanced the activity of NK cells and macrophages [218].

Chronic infection with the intracellular protozoan parasites *T. gondii* and *B. jellisoni* induced significant resistance to the development of leukemia in AKR mice [204]**.**

#### Lymphomas

Lymphomas are a type of cancer that starts in the lymphatic system, which is part of the body’s immune system. They account for 3–4% of all cancers and are the seventh-most common form. In children, they are the third most common cancer. Lymphomas occur more frequently in developed countries than in developing countries [219]. There are two main types of lymphoma: non-Hodgkin, which makes up about 90% of cases, and Hodgkin lymphoma. Non-Hodgkin lymphoma has around 40 subtypes, each with different growth rates and responses to treatment [220]. Treatment options include chemotherapy, radiation therapy, and monoclonal antibodies. In some cases, stem cell transplant with strong chemotherapy may be necessary if the lymphoma has recurred or is likely to recur in the future [221].

#### Antineoplastic activity of helminth parasites against lymphomas

As regards helminth parasites’ anti-neoplastic activity against lymphoma, it has been demonstrated that macrophages from *T. spiralis-*infected mice had a powerful cytotoxic effect on syngeneic EL-4 lymphoma cells in vitro [222].

#### Antineoplastic activity of protozoan parasites against lymphomas

Protozoan parasites such as *T. cruzi* exhibited an anti-tumor activity against lymphoma. Infection of mice with *T. cruzi* CH4 strain resulted in the inhibition of growth and metastasis of solid L5178Y-R lymphoma transplanted to BALB/c mice. This effect was more pronounced when the tumor was transplanted during the sub-acute phase of *T. cruzi* infection, as opposed to the acute phase. The mechanism behind this anti-tumor activity appears to involve the production of cytotoxic/cytostatic substances and cross antibodies against both the parasite and the tumor antigens [223].

Regarding the *Plasmodium parasite*, VDC886, a complex formed by conjugating recombinant malaria protein rVAR2 with a hemiasterlin analog (KT886), induced in vitro strong cytotoxicity to MyLa2059 T-cell lymphoma and significantly inhibited the growth of non-Hodgkin’s lymphoma (Karpas 299) in the murine model [90].

The inhibitory effect of *T. gondii* on lymphoma growth has been demonstrated in cell culture and mouse models. As early as 1986, formalin-fixed *Toxoplasma* organisms were found to have a potent anti-tumor effect on EL4 lymphoma in mice, leading to a significant suppression of tumor growth and an increase in lifespan. This effect was more evident compared to Bacillus Calmette-Guerin denoting its powerful immunostimulant effect [224]. Another study reported that the in vivo treatment with *Toxoplasma* lysate antigen significantly enhanced the cytotoxicity of feline spleen cells against feline leukemia virus-producing lymphoma cells [225]. In the same line, *T. gondii* lysate antigen or its antigenic component, *Toxoplasma* lysate-Ig8 also demonstrated significant inhibition of tumor growth induced by 20-methylcholanthren in the murine models [226–228] and induced cytotoxic activity on the YAC-1 murine lymphoma cell line [226].

Additionally, both live *T. gondii* and *Toxoplasma* lysate antigen reduced the multidrug resistance of mouse lymphoma cell lines (Mdr L 5718 and Par 5718) making them more susceptible to cytostatic drugs by enhancing drug accumulation through reducing the activity of ATP-dependent efflux pump [107].

#### Multiple myeloma

Multiple myeloma is a type of hematological bone marrow malignancy. It arises from the clonal proliferation of defective plasma cells, and this causes harmful effects on multiple organs from the deposition of monoclonal paraproteins. It is currently viewed as a treatable but incurable condition [229]. First-line therapy usually involves proteasome inhibitors or immunomodulatory drugs. Because of side effects, these medications are usually used in combination with high-dose corticosteroids. Monoclonal antibody therapies are also now available but can also interfere with blood grouping. In fit patients, autologous stem cell transplantation is recommended to consolidate response to the first-line treatment [230]. Regarding helminth parasites’ anti-neoplastic activity against multiple myeloma, Tsocheva et al*.* [116] discovered that biologically active substances isolated from the tissues of *F. hepatica* had a slight inhibitory effect on myeloma cell culture proliferation.

Interestingly, myeloma-associated antigens have been detected in *T. spiralis* parasite. Cross-immune responses between antigens of the myeloma cell SP2/0 versus positive sera to *T. spiralis*, and antigens of *T. spiralis* versus positive sera to myeloma cell SP2/0 were detected using *T. spiralis* and myeloma-specific enzyme-linked immunosorbent assays (ELISA). The study identified that the main component of the myeloma-associated antigens was tropomyosin, which is a protein found in *T. spiralis* myofibrils. Tropomyosin is an acidic protein that is located on the surface and consists of 284 amino acids. Interestingly, when mice were immunized either with tropomyosin or crude antigen of *T. spiralis*, it resulted in an anti-tumor effect. This was demonstrated by a significant increase in CD4^+^, CD8^+^ T lymphocytes, and CD19^+^ B lymphocytes. These findings suggest that tropomyosin may have a role in triggering cross-protective immunity [231]. Furthermore, immunoscreening of a *T. spiralis* cDNA expression library identified antigen genes responsible for the anti-tumor activity of *T. spiralis*. They discovered that the antisera raised against a novel gene product of *T. spiralis* cross-reacted with Sp2/0 myeloma cells. The sequencing analysis revealed that the fragment was 569 bp long and contained an open reading frame. It was predicted that the full-length gene encoded 136 amino acids. This gene known as TS2, had four potential N-Arg dibasic convertase (Nardilysin) cleavage sites, one peptide C-terminal amidation site, and one glycosaminoglycan attachment site. Bioinformatic analysis predicted six antibody epitopes [232]. Moreover, in another study using suppression-subtractive hybridization, researchers were able to identify genes that were expressed differently between tumor cells from *T. spiralis*-infected and non-infected mice. The results indicated that genes encoding RpL41, NKTR, Rbbp4, and ANXA2 were enriched and considered possible proteins responsible for the anti-tumor activity of *T. spiralis* [233]. Therefore, further investigations are needed to explore the potential use of these novel antigen genes as an anti-myeloma vaccine.

#### Mastocytoma

Mastocytoma is a rare tumor of mast cells, which are derived from myeloid stem cells and found in connective tissues, mainly in the skin and mucosal linings [234]. The clonal proliferation of neoplastic mast cells can cause localized and systemic manifestations, known as ‘mastocytosis’ and classified as a myeloproliferative disorder in the WHO classification of tumors of hematopoietic and lymphoid tissues [235]. In humans, advanced mast cell neoplasms are rare and have a poor prognosis, with limited treatment options available [236].

#### Antineoplastic activity of helminth parasites against mastocytoma

Activated macrophages from mice infected with *T. spiralis* inhibited allogeneic P815 tumor cells, a murine mastocytoma cell line [222].

#### Antineoplastic activity of protozoan parasites against mastocytoma

The protozoan parasite *T. gondii* induced an anti-neoplastic activity against mastocytoma. In an in vitro study, *T. gondii* lysate antigen demonstrated strong cytolytic activity on P-815 cells [226].

### Connective tissue and bone cancers

Table 6a, b shows the in vitro and in vivo studies of the anti-neoplastic activity of different parasites against connective tissue and bone tumors (sarcomas and bone tumors).

#### Sarcomas

Sarcomas are malignant connective tissue tumors. Fibrosarcoma is a type of soft tissue sarcoma that originates from fibroblasts and typically starts in the fascia and tendons of soft tissue but can also occur in bones. Fibrosarcomas can be divided into two types: infantile or congenital fibrosarcomas which rarely metastasize, and adult-type fibrosarcomas which are highly malignant. Treatment for fibrosarcoma usually involves a combination of surgery, chemotherapy, and radiation. However, fibrosarcoma is resistant to chemotherapy and has a high rate of recurrence [237–239]. Therefore, it is important to find new methods to improve the treatment of this tumor. Recent data suggest that immunotherapy may be effective in treating soft tissue sarcomas. This raises hope for novel treatments that target the tumor microenvironment [240].

#### Antineoplastic activity of helminth parasites against sarcomas

Both in vivo and in vitro studies have shown that certain helminth parasites can hinder sarcoma growth. For instance, in the murine model, *S. mansoni* infection-induced resistance against transplantable sarcoma 180 ascites tumor cells [241].

Live hydatid cyst protoscolices have been found to inhibit proliferation and induce cell death in WEHI-164 fibrosarcoma cells in vitro denoting their strong anti-cancer activity [242]. Similarly, *T. spiralis* crude antigens have demonstrated in vitro antiproliferative effect against a murine sarcoma cell line (S180) [104]. Moreover, in vivo testing has revealed that infection with *T. spiralis* or treatment of sarcoma-bearing mice with crude extracts of adult and newborn larvae of *T. spiralis* can inhibit tumor growth under selected conditions of larval dose and tumor challenge interval [104, 243, 244]. Additionally, *T. canis* egg antigen has been found to inhibit tumor growth in the WEHI-164 fibrosarcoma BALB/c mouse model [245].

#### Antineoplastic activity of protozoan parasites against sarcomas

Sphingolipids, a member of the lipid family, play a role in various cellular processes such as cell growth, adhesion, migration, senescence, and programmed cell death. They have shown potential for regulating cell growth and inducing apoptosis in cancer cells, making them a promising target for cancer therapy [246]. Studies have demonstrated that *L. donovani* sphingolipid-1, a component of sphingolipids has significant cytotoxic effects on murine sarcoma 180 cells in vitro*.* It altered mitochondrial homeostasis and activated caspases in a dose- and time-dependent manner [247, 248]. In a sarcoma 180 murine model, it improved the survival rate of tumor-bearing mice and reduced tumor growth in a dose-dependent manner. It also inhibited angiogenesis and regulated the expression of matrix metalloproteinases and cytokines making it a potential therapeutic for sarcoma [248].

Several studies demonstrated the anti-sarcoma effect of *T. gondii*. Chronic infection with *T. gondii* significantly induced resistance to the development of sarcoma 180 in Swiss mice [204]. Similarly, *T. gondii* antigen has been found to inhibit WEHI-164 fibrosarcoma growth in BALB/c mice [245].

Interestingly, immunotherapy of fibrosarcoma-bearing mice was induced using DCs that were treated with protein components of *T. gondii*. Such treatment significantly inhibited tumor growth, improved the survival of the mice, and revealed that the protein components of *Toxoplasma* were able to increase the capability of DCs. This induced a significant increase in the activity of cytotoxic T cell-mediated anti-tumor response and the infiltration of CD8^+^ T cells into the tumor microenvironment [249, 250]. Furthermore, treatment of mice bearing sarcoma-180 tumors model with *T. gondii* lysate antigen reduced tumor size and decreased the expression of CD31, an angiogenesis marker within the tumor tissue [251]. Surprisingly, in an in vitro experiment, three live protozoan parasites were examined for their anti-cancer effects on WEHI-164 fibrosarcoma cells. These parasites were *T. gondii* tachyzoites, *T. vaginalis* trophozoites, and *L. major* promastigotes. It was noticed that only *T. gondii* showed a decrease in fibrosarcoma cell proliferation and an increase in cell lysis in the cell culture medium. It is interesting to note that although both *T. gondii* and *L. major* are intracellular parasites, they have different effects on fibrosarcoma cells [252].

The anti-tumor activity of *Eimeria* spp*.* has been demonstrated in a murine model of sarcoma (S180). Both native and recombinant soluble protein extracts of *E. tenella* have shown potent immunostimulatory effects, significant anti-tumor activity, and potent IL-12-inducing properties. This was evidenced by a significant reduction in tumor size, increased mice survival, and cure rates of over 80%. Therefore, *E. tenella* protein could be considered a new type of innate immunity stimulator and may be more therapeutically beneficial than others due to its lack of toxicity even at very high doses [253]. Likewise, chronic infection with *B. jellisoni* also *s*ignificantly induced resistance to sarcoma 180 development in Swiss mice [204].

#### Bone tumors

Primary bone tumors are classified as either benign such as osteoma, osteochondroma, or giant cell tumor of bone among others, or malignant such as osteosarcoma, Ewing sarcoma, chondrosarcoma, fibrosarcomas, and others. Bone tumors are challenging to prevent and treat and current methods such as surgery, radiotherapy, and chemotherapy have severe side effects. This highlights the need for new approaches [254, 255].

Interestingly, a study found that antigens from hydatid cysts, specifically the crude extract of the laminated layer antigens, reacted with the serum of different bone cancer patients. Two specific antigens, 43 kDa, and 70 kDa, were identified. This suggests that there may be a similarity in the immunogenic antigens between hydatid cysts and different bone cancers [256].

In another study, anti-TPD52 antiserum was developed against the cross-antigen gene tumor protein D52 (TPD52) found in human osteosarcoma and *T. spiralis*. This antiserum showed both in vitro and in vivo anti-osteosarcoma activity. It inhibited the proliferation of human osteosarcoma MG-63 cells and induced apoptosis in a dose-dependent manner. In a mouse model of osteosarcoma, the anti-TPD52 antiserum was more effective than the anti-*T. spiralis* antiserum and enhanced immunity as demonstrated by increased levels of IFN-γ, TNF-α, and IL-12 in the serum, without causing any histopathological damage. Thus, the anti-TPD52 antiserum significantly inhibited osteosarcoma growth by inducing apoptosis and enhancing immunity [54].

### Skin cancers

Table 7a, b shows the in vitro and in vivo studies of the anti-neoplastic activity of different parasites against skin cancers (Melanoma and Merkel Cell Carcinoma).

#### Melanoma

Melanoma, a deadly form of skin cancer, is caused by abnormal proliferation of melanocytes. It affects the deep layers of the skin and can easily spread to other tissues. The main cause of melanoma is believed to be exposure to ultraviolet radiation. Recent research has shown that melanoma is not a single disease but a group of diseases with different genetics, histopathological, and clinical characteristics [257, 258]. One major challenge in treating melanoma is drug resistance [259]. However, there have been promising advancements in combined therapy regimens, particularly with the use of immunotherapy and new therapeutics [260].

#### Antineoplastic activity of helminth parasites against melanoma

Finding new therapeutic strategies for treating melanoma is an inspiring opportunity for research. According to numerous studies, certain worms and protozoa have shown anti-melanoma activity. For example, *E. granulosus*, a worm parasite contains compounds that have been found to exhibit anti-tumor activity. In a study, hydatid cyst fluid or excretory–secretory antigen of protoscolices of *E. granulosus* inhibited melanoma cancer growth in C57 black mice [261]. Similar results were obtained in the other studies, in which mice-bearing melanoma cancer were treated using live protoscolices, hydatid cyst fluid, or a fraction of cyst fluid. Significant inhibition of tumor growth was observed, along with differences in the levels of measured cytokines such as IL-2, TNF-α, IFN-γ, and IL-4 [262, 263]. In vitro, studies demonstrated that hydatid cyst fluid and AgB from hydatid cyst fluid inhibited the proliferation and promoted apoptosis of the A375 human melanoma cancer cells and B16.F10 melanoma cancer cell line, respectively. This was achieved by increasing the mRNA expression of the pro-apoptotic gene BAX, Caspase-9, and Caspase-3, and decreasing the mRNA expression of the anti-apoptotic gene BCL2 [264, 265]. Additionally, recombinant *E. granulosus* Kunitz-type protease inhibitor (EgKI-1) inhibited the growth and migration of melanoma cells (CJM) in a dose-dependent manner [186].

In another study, the synthetic *T. crassiceps* cysticerci-derived peptide GK1 showed anti-melanoma activity in a mouse melanoma model of B16-F10 melanoma cells. GK1 is an 18-amino acid peptide that boosts the immune response. The treatment with GK1 significantly prolonged the survival time of melanoma-bearing mice, slowed down tumor growth, and increased tumor necrosis. This suggests that GK1 could be used as a primary or adjuvant component for cancer treatment by stimulating pro-inflammatory cytokines [266]. Interestingly, when GK1 was combined with anti-programmed death ligand 1 therapy in the mouse melanoma model of B16-F10-luck melanoma cells, the survival time was even longer compared to each treatment alone. Additionally, the combination treatment led to a decrease in TNF-α, IL-4, IL-5, IL-6, and IL-10 serum levels, indicating the beneficial effects of GK1 [267].

*Trichinella spiralis* has been found to have an anti-cancer activity against melanoma. In a study, infection of B6D2F1/J mice with *T. spiralis* followed by its challenge with viable B-16 melanoma cells developed strong anti-neoplastic activity. None of the infected mice showed any signs of neoplasia or developed detectable tumors, while all of the control mice did [268]. In another study, oral infection of melanoma-bearing C57B1/6 with *T. spiralis* larvae showed a significant decrease in tumor growth and lung metastases. This was attributed to changes in cytokine regulation, specifically in the levels of CXCL9, CXCL10, and CXCL13 [269]. Similarly, chronic *T. spiralis* infection also induced an impressive reduction in tumor size with a longer tumor induction interval in a mouse melanoma model [270, 271]. Moreover, the ESPs of the first larval stage of *T. spiralis*, which is released during the chronic stage of infection, inhibited the survival and induced mild apoptosis of mouse (B16) melanoma cells [270].

#### Antineoplastic activity of protozoan parasites against melanoma

In addition to worms, protozoan parasites like the pathogenic/free-living amoeba *Acanthamoeba castellanii* (*A. castellanii*), *L. donovani*, *T. cruzi*, *Plasmodium* spp., *T. gondii*, *N. caninum*, and *Babesia microti* (*B. microti*) have also shown anti-melanoma activity. An in vitro study demonstrated that *Leishmania* sphingolipid-1 (LSPL-1) has cytotoxic effects and induces apoptosis in murine and human melanoma cells (B16F10 and A375 cell lines) by regulating the mitochondrial death pathway and generating reactive oxygen species. LSPL-1 up-regulated the expression of p53, p21, NOXA, and PUMA which are involved in p53-mediated apoptosis and bind with Bcl-2 and Bax to induce apoptosis in a concentration- and time-dependent manner [247, 272, 273]. In a B16F10 cell-induced xenograft melanoma murine model, LSPL-1 improved survival time, elicited an immunostimulatory response, and significantly reduced tumor volume in a dose-dependent manner. It also reduced the metastatic flare of angiogenic factors such as VEGF, Ang-2, and CD34 without any deleterious effects on the murine model. Therefore, LSPL-1 can be considered a promising therapeutic of parasitic origin against melanoma and its metastatic properties [247].

*Acanthamoeba castellanii* is one of the most common protozoa found in the environment. In humans, it can cause diseases such as keratitis, which can lead to corneal ulceration or blindness, and granulomatous amoebic encephalitis which is often seen in immunosuppressed patients [274]. An in vitro study has shown that viable *A. castellanii* and its parasite lysates can induce extensive cytolysis of human melanoma (OCM-1) and murine melanoma (D5.1G4) cells. Additionally, intratumoral injection of viable parasites or parasite lysates into progressively growing subcutaneous melanomas in a mouse model resulted in 83% and 53% reductions, respectively, in tumor masses compared with untreated controls [275].

As regards *T. cruzi*, a recombinant protein named J18, which is based on *T. cruzi* surface molecule gp 82 fused to glutathione-*S*-transferase (GST) induced cell death and apoptosis in Tm5 melanoma cells in vitro. Moreover, melanoma-bearing C57BL/6 mice treated with J18 at the site of tumor cell inoculation developed smaller tumors and had longer survival compared to control mice [42].

In another study, immunization with a plasmid encoding survivin, a tumor-associated antigen, along with the administration of systemic recombinant *T. cruzi* Calreticulin (r*Tc*CRT), had a synergistic effect on inhibiting tumor growth and inducing humoral anti-r*Tc*CRT immunity in a B16-F10 melanoma murine model [276]. In the same line, a further study has shown that *T. cruzi* derived Toll-Like Receptor (TLR) agonists, such as glycoinositolphospholipids and CpGs oligodeoxynucleotides, are effective immunological adjuvants. They have been found to induce significant protective immunity and cause a significant delay in the growth of B16F10 melanoma cells expressing a cancer-testis antigen (NY-ESO-1) in a murine model. These agonists also stimulate the production of IFN-γ by CD4^+^ T cells. Additionally, CpGs oligodeoxynucleotides derived from the parasite but not glycoinositolphospholipids triggered a potent IFN-γ response by CD8^+^ T lymphocytes [277].

Interestingly, the use of recombinant non-pathogenic clone of *T. cruzi* as a vaccine vector expressing a cancer-testis antigen (NY-ESO-1), a cancer vaccine candidate, which resulted in strong and long-term T-cell-mediated immunity and solid protection against melanoma in mice [278]. Additionally, its combination with blockade of Cytotoxic T Lymphocyte Antigen 4 resulted in a significant therapeutic response. This combination enhanced the frequency of NY-ESO-1-specific effector CD8^+^ T cells producing IFN-γ and promoted lymphocyte migration to the tumor infiltrate, ultimately controlling the development of an established melanoma in the murine model [279].

Research studies on the anti-cancer effects of the *Plasmodium* parasite on melanoma have shown promising results. Animal studies have demonstrated that infection with the 17XL strain of *P. yoelii* significantly delayed the occurrence of tumors and inhibited the growth of melanoma in the B16F10 tumor cells melanoma model [280]. Additionally, VDC886, a hemiasterlin analog (KT886) conjugated with the refined malaria protein VAR2, has shown anti-cancer activity in various types of cancer, including strong cytotoxicity to C3 human and B16 murine melanoma cells [90]. Furthermore, malaria-mimicking erythrocyte nanoparticles (MMENPs), a lipid catcher-tag conjugation system for the functionalization of erythrocyte membrane-coated drug carriers with recombinant VAR2 CSA (rVAR2), loaded with the anti-cancer drug docetaxel (DTX) have been shown to specifically target and kill melanoma cells in vitro*.* They also demonstrate effective targeting and therapeutic efficacy in a xenografted melanoma animal model [281]. Similarly, a study developed a recombinant VAR2CSA peptide (rVAR2)-modified activated platelet-mimicking nanoparticles (rVAR2-PM/PLGA-ss-HA) by coating the surface of disulfide-containing biodegradable PLGA conjugate nanoparticles (PLGA-ss-HA) with an activated platelet membrane. Loading these nanoparticles with the chemotherapy drug, DTX, exhibited a significant synergistic and active tumor-targeting, anti-neoplastic effect against primary and lung metastasis in a mouse model of melanoma (B16F10). It significantly reduced the volume and weight of the primary tumor, decreased the number of lung metastasis nodules, and induced a high level of tumor cell apoptosis. This synergistic active targeting of tumor cells depends on the activated platelet membrane and chondroitin sulfate receptors on the tumor surface, regardless of their location. Once these nanoparticles are endocytosed and become inside the tumor cells, they release, DTX, resulting in the death of tumor cells. This study suggests that the rVAR2-decorated activated platelet-targeting nanoparticles with controlled drug release could be an effective strategy for treating both primary and metastatic cancer [282].

Several studies have shown that *T. gondii* effectively inhibits melanoma growth. Syngeneic C57BL/6 mice infected with *T. gondii* on the same day of B16.F10 melanoma cells implantation experienced a significant suppression of melanoma growth and strong systemic suppression of angiogenesis [283]. Similarly, treatment of B16 melanoma-bearing mice with *T. gondii* excretory–secretory antigens suppressed tumor growth and led to a decrease in CD4^+^ CD25^+^ Treg cells and an increase in NK cells [284]. Additionally, two protein fractions A1 and C14 of *Toxoplasma* tachyzoite antigens were identified as potent activators of DCs, with A1 proving to be more effective than C14 in inducing an immune response inducing slowing tumor growth and prolonged mice survival time [285].

The use of non-pathogenic live protozoan parasites as anti-cancer therapeutic approaches has gained global attention. Live organisms have been found to elicit a much stronger anti-tumor effect than dead ones. Several studies have shown that intratumoral injection with mutant *T. gondii* strain can enhance anti-tumor immunity. In one study, treatment of melanoma-bearing mice with the non-replicating uracil auxotroph strain of *T. gondii* (cps strain) effectively halted tumor growth and induced a systemic anti-tumor immune response, while also modifying the tumor microenvironment [286]. Similar results were obtained with intratumoral administration of attenuated *T. gondii* mutants lacking two lactate dehydrogenases (ME49 *Δldh1–Δldh2*), which repressed the growth of established tumors and inhibited lethal tumor development in a mouse melanoma model [287]. Furthermore, intratumoral injection of attenuated RH-ΔGRA17 mutant *T. gondii* tachyzoites boosted anti-tumor immunity and the effectiveness of checkpoint inhibition therapy. This significantly extended the survival of mice and suppressed tumor growth in both targeted and distal tumors in pre-clinical mouse models of melanoma tumors with better treatment outcomes compared with monotherapy with *Toxoplasma* [95].

Interestingly, a study investigated the reactivity of different antibodies with mouse melanoma cells using flow cytometry. Surprisingly, only anti-*T. gondii* antiserum reacted strongly with mouse melanoma cells, suggesting the possibility of shared antigens between *Toxoplasma* and melanoma cells. Antibodies against *T. vaginalis*, hydatid cyst fluid, and protoscolice antigens did not show significant reactivity with these cells [53].

For the other coccidian parasite, *N. caninum*, a study in a C57BL/6 mouse melanoma model showed that intratumoral and distal subcutaneous injections of parasite tachyzoites had a significant inhibitory effect on tumor growth, resulting in over 50% of tumor cells being dead. This treatment also increased the infiltration of CD8^+^ T cells and macrophages, as well as the mRNA expression levels of IL-12, IFN-γ, IL-2, IL-10, TNF-α, and PD-L1 in the tumor microenvironment. In the spleen of tumor-bearing mice, IL-12 and IFN-γ levels were also increased. Overall, *N. caninum* inhibits B16F10 melanoma by activating strong immune responses and directly destroying cancer cells [288].

Since 85% of advanced-stage melanoma patients have lung metastases, which is the leading cause of their death, a study was conducted to investigate the potential of intranasal administration of *N. caninum* or engineered *N. caninum* to secrete human interleukin IL-15/IL-15Rα in the microenvironment, in a syngeneic C57BL6 mouse model of B16-F10 melanoma lung metastases. The study demonstrated that the engineered *N. caninum* was more effective than wild *N. caninum* in preventing further progression of lung metastases. In mice treated with the engineered parasite, only 0.08% of the lung surface had metastases, compared to 4.4% in wild-type *N. caninum* treated mice and 36% in untreated mice. The control of tumor development was associated with a significant increase in NK cells, CD8^+^ T cells, and macrophages in the lung. Additionally, the macrophages showed polarization toward an anti-tumoral M1 phenotype. This study demonstrates the potential of engineered *N. caninum* as a safe and effective immunotherapeutic approach for the treatment of metastatic solid cancers, which currently have limited treatment options [289].

*Babesia microti*, an intraerythrocytic protozoan parasite, is transmitted through the bite of an infected Ixodes tick. It primarily infects small rodents but can also infect humans which are dead-end hosts. Most infections are asymptomatic and clinical recovery usually occurs [290]. It has been shown in an in vivo study that mice infection with *B. microti* inhibited the growth of melanoma in tumor-bearing mice and prolonged their survival time. *Babesia microti* stimulated the immune system and induced polarization of immunosuppressive M2 macrophages to pro-inflammatory M1 macrophages. This led to an increase in the number of macrophages, CD4^+^ T cells, the proportion of CD4^+^ T cells, and M1 macrophages as well as inhibition of angiogenesis in the tumor microenvironment [291].

#### Merkel-cell carcinoma (MCC)

Merkel-cell carcinoma is a rare and aggressive form of skin cancer that affects about three people per million in the population. This cancer affects the epidermis and often spreads to lymph nodes and organs. Risk factors for MCC include exposure to ultraviolet light, weakened immune system, age, and infection with the Merkel-cell polyomavirus. Surgical excision with a safety margin is the primary treatment for early cases, but it often recurs. Advanced MCC carcinoma may respond to chemotherapy, but the responses are usually temporary. Immunotherapies have shown promise in treating advanced-stage or chemotherapy-resistant MCC [292, 293].

*Neospora caninum* displayed a highly effective and non-toxic anti-cancer activity against MCC. It could infect, multiply, and destroy human tumor Merkel cells [The WaGa (RRID:CVCL E998)]. In a model of NOD/SCID mice-bearing human MCC, intratumoral injection of *N. caninum* showed strong efficacy in destroying cancer cells and triggering potent anti-tumor immune responses, resulting in a threefold reduction in tumor size. This study highlights the potential clinical application of using *N. caninum* as an oncolytic protozoan in human medicine [294].

### Miscellaneous cancers

Table 8a–e shows the in vitro and in vivo studies of the anti-neoplastic activity of different parasites against miscellaneous cancer subtypes (Ovarian Cancer, Cervical Cancer, Prostate Cancer, Neuroglial and Neural Crest Cancers, Thymoma and Head and Neck Squamous Cell Carcinoma).

#### Gynecological cancers

**Ovarian cancer** Ovarian cancer is the leading cause of death among women with gynecological cancers and the fifth most common cause of death among women in general [295]. The standard treatment includes surgery and platinum-based chemotherapy, but there has been increasing use of anti-angiogenic Bevacizumab and Poly(ADP-ribose) polymerase (PARP) inhibitors in the past decade [296]. Unfortunately, most of the cases are diagnosed at an advanced stage, which leads to poor outcomes. Additionally, there is a high rate of recurrence after the initial treatment with these relapsed cases being less curable and more likely to experience treatment failures [297, 298]. Therefore, there is a pressing need for new and effective treatment options based on a better understanding of the molecular characteristics of this cancer [299].

#### Antineoplastic activity of helminth parasites against ovarian cancer:

A research study has shown that helminth parasites, specifically recombinant *T. solium* Calreticulin (r*Ts*CRT), have demonstrated anti-cancer activity against ovarian cancer. An in vitro study has shown that r*Ts*CRT inhibits the growth of the ovarian cancer cell line (SKOV3) by interacting with scavenger receptors in a dose-dependent manner [75].

#### Antineoplastic activity of protozoan parasites against ovarian cancer:

For protozoan parasites, cps is an avirulent non-replicating uracil auxotroph strain of *T. gondii* that has shown promising anti-neoplastic activity. When administered intraperitoneally, cps triggers the rejection of established ID8-VegfA tumors, which is an aggressive xenograft model of ovarian carcinoma. Cps preferentially invades tumor-associated antigen-presenting cells in the ovarian carcinoma microenvironment and restores their ability to trigger potent anti-tumor CD8^+^ T-cell responses. The therapeutic benefit of cps treatment relies on the expression of IL-12, but it is unexpectedly independent of MyD88 signaling. Thus, applications of cps as an immunotherapeutic agent might be helpful to reawaken natural immunity in the immunosuppressive microenvironment of some solid tumors [300]*.* Additionally, secretion of rhoptry and dense granule effector proteins by the non-replicating *T. gondii* uracil auxotrophs activated anti-tumor immunity through host responses involving CD8α^+^ DCs, the IL-12/IFN-γ Th 1 axis, as well as CD4^+^ and CD8^+^ T cells and reversed immune suppression in the tumor microenvironment [301].

Interestingly, in an in vitro experiment, extracts of *T. gondii* and *L. major* inhibited the proliferation of susceptible (A2780) and resistant (A2780-CP) ovarian cancer cell lines to cisplatin due to their complex excretion/secretion antigens [302].

**Cervical cancer** Worldwide, cervical cancer is both the fourth-most common type of cancer and a contributor to cancer-related deaths in women. Infection with helminths has been linked to an increase in human papillomavirus (HPV) prevalence, and persistent HPV infection is responsible for nearly all new cervical cancer cases [303].

#### Antineoplastic activity of helminth parasites against cervical cancer:

Despite the increased incidence of cervical cancer in helminth endemic areas, several in vitro and in vivo studies demonstrated the anti-neoplastic activity of certain helminths against cancer cervix. In vitro studies showed that both recombinant *E. granulosus* Kunitz-type protease inhibitor (EgKI-1) and recombinant *T. solium* Calreticulin (rTsCRT) either alone or in combination with 5-fluorouracil inhibited the growth and migration of cervical epithelial adenocarcinoma (HeLa) cells (HPV18-positive epithelial adenocarcinoma cells) in a dose-dependent manner. This was achieved through disruption of the cell cycle and triggering of apoptosis by EgKI-1 whereas, rTsCRT interacted with scavenger receptors [75, 186]. Also, *Nippostrongylus brasiliensis* (*N. brasiliensis*) third-stage larvae (L3), a rodent hookworm parasite, reduced cell migration of HeLa, Ca Ski (HPV16 positive epithelial epidermoid carcinoma cells) and C33-A (HPV negative epithelial carcinoma cells) cervical cancer cell lines. *N. brasiliensis* L3 antigen decreased HPV type 16 (HPV16) pseudovirion internalization and reduced expression of vimentin and N-cadherin which are associated with the transition of epithelial cells to a more invasive and metastatic mesenchymal cell type. Moreover, in vivo testing revealed that infection with *N. brasiliensis* L3 can systemically alter genital tract mesenchymal markers in a manner that may impair cervical cancer cell progression. This was demonstrated by a significant reduction in vimentin expression within the female genital tract in a murine model [61].

As for the other helminth *T. spiralis*, biologically active substances isolated from the livers of *T. spiralis*-infected rats showed a mild inhibiting effect on the growth of human cervical carcinoma HeLa cells, although they had a strong inhibitory effect on Graffi myeloid tumor cells [304].

#### Antineoplastic activity of protozoan parasites against cervical cancer:

Numerous in vitro studies have demonstrated the anti-neoplastic activity of *T. vaginalis* on cervical cancer cell lines. In one study, the culture supernatant of *T. vaginalis* inhibited the growth of HeLa cervical cancer cells by inhibiting proliferation and promoting apoptosis [305]. In another study, live *T. vaginalis* isolate T016 and its excretory–secretory product induced apoptosis of human cervical cancer cells (SiHa cells), and vaginal epithelial cells (MS74 cells) in a parasite-dose-dependent manner through the induction of mitochondria-dependent apoptosis via the dissociation of Bcl-xL/Bim and Mcl-1/Bim complexes [306]. This is consistent with other studies that have implicated the involvement of the mitochondria-dependent intrinsic apoptotic pathway through an NF-κB-regulated, mitochondria-mediated pathway involving the activation of reactive oxygen species [307, 308] and the promotion of endoplasmic reticulum stress-mediated mitochondrial apoptosis via the IRE1/ASK1/JNK/Bcl-2 family protein pathways [308].

#### Prostate cancer

Prostate cancer is a major health concern for men with poor prognosis for advanced castration-resistant prostate cancer. In the early stages, prostate cancer is symptomless. However, as the tumor grows, it can cause erectile dysfunction, blood in the urine or semen, and difficulty in urinating [309, 310]. In some cases, prostate cancer can spread to other parts of the body, particularly the bones and lymph nodes causing severe pain, leg weakness, or paralysis, and eventually leading to death [311].

Research studies have shown that certain protozoan parasites, including *T. vaginalis*, *Plasmodium*, and *T. gondii*, can induce an outstanding anti-neoplastic activity against prostate cancer. In vitro experiments have demonstrated that the culture supernatant of *T. vaginalis* inhibited the proliferation and promoted apoptosis of PC-3 and DU145 prostate cancer cell lines cells, through up-regulation of the antiproliferative molecule p21 and down-regulation of the anti-apoptotic molecule Bcl-2 [312]. Similarly, the refined malaria protein rVAR2–drug conjugates, recombinant rVAR2-DT fusion protein [genetically fused the cytotoxic domain of Diphtheria toxin (DT388) to rVAR2], and VDC886 [hemiasterlin analog (KT886) conjugated to rVAR2] demonstrated strong cytotoxicity to human prostate cancer cells (PC-3). Additionally, it induced significant inhibition of the growth of the metastatic castration-resistant prostate cancer cell line, PC-3 in the mouse tumor xenograft model. Importantly, these conjugates were found to be safe with no signs of toxicity to the kidney or liver tissues in the murine animal model [90]. Also, *T*. *gondii* tachyzoites showed an inhibitory effect on DU-145 prostate cancer cell line proliferation [105].

#### Neuroglial and neural crest cancers

Table 8c shows the in vitro and in vivo studies of the anti-neoplastic activity of different parasites against neuroglial and neural crest cancers (Gliomas, Medulloblastoma, Ependymoblastoma, and Neuroblastoma).

#### Gliomas

Gliomas are the most common type of primary brain tumor in adults, accounting for 40% of all central nervous system tumors. Treatment of gliomas requires a multidisciplinary approach involving surgery, radiotherapy, and chemotherapy. The choice of treatment modality depends on the type of glioma, which is determined by clinical-histological features, molecular evidence, and anticipated prognosis. However, progress in improving survival outcomes for gliomas is still limited and challenging. Therefore, it is important to explore novel therapies [313, 314].

#### Antineoplastic activity of helminth parasites against gliomas:

The anti-cancer effects of *T. spiralis* have been studied in gliomas. Liu and colleagues demonstrated that *T. spiralis* infection had anti-neoplastic activity against glioma in BALB/C mice, and that effectiveness was dependent on the parasite load [315].

#### Antineoplastic activity of protozoan parasites against gliomas

A research group discovered that *T. vaginalis* purine nucleoside phosphorylase (*Tv*PNP) improved the cleavage of F-araAMP or deoxyadenosine analogs used for cancer therapy. In a human glioma D54 xenograft murine model stably transduced with *Tv*PNP (D54-*Tv*PNP) showed significant anti-tumor effects when F-araAMP was administered systemically or intratumorally, with catalytical efficiency 25 times greater than *Ec*PNP [316].

In another study, VDC886, a refined malaria protein conjugated with a cytotoxic compound, exhibited strong cytotoxicity to glioma cells in vitro [90]. In orthotopic and subcutaneous models of mouse glioma (GL261), immunotherapy with the non-lethal *P. yoelii*, either alone or in combination with radiotherapy, induced synergistic anti-tumor effects. The combination therapy significantly slowed tumor growth and prolonged the life span of tumor-bearing mice more than monotherapy. This was achieved by up-regulating the perforin-expressing effector CD8^+^ T cells and down-regulating the myeloid-derived suppressor cells. Most importantly, the combination therapy was able to cure approximately 70% of gliomas. Therefore, it is important to consider introducing this combination therapy to clinical trials for the treatment of glioma [171].

Similarly, the *T. gondii* lysate antigen has been found to have anti-neoplastic activity against gliomas in both in vitro and in vivo studies. In the in vitro study, it was observed that the *Toxoplasma* lysate antigen suppressed the proliferation and invasion of two human glioma cells, U373MG and U87MG, in a dose-dependent manner. At high concentrations, *Toxoplasma* lysate antigen also induced apoptosis in the glioma cells. In the in vivo study, the *Toxoplasma* lysate antigen decreased tumor growth in athymic nude mice that were transplanted with human malignant glioma cells. Additionally, the addition of Quil-A to the *Toxoplasma* lysate antigen treatment significantly enhanced its anti-tumor effects [317].

#### Medulloblastoma

Medulloblastoma is the most common malignant brain tumor in children, originating from the cerebellum. Current treatment involves surgery, chemotherapy, and radiation therapy. This multimodal approach has improved long-term survival rates for 70%–80% of patients. However, approximately 30% of patients still do not survive, and those who do, often experience long-term side effects that affect their quality of life [318].

One of the challenges in treating medulloblastoma is the lack of T-cell infiltration and a supportive tumor microenvironment which hinders immunotherapy. However, a study by Nguyen et al. [319] showed that in a mouse model of medulloblastoma, *T. gondii* infection reduced tumor incidence. This infection attracted T cells to the brain tumors and transformed the non-supportive tumor microenvironment into a supportive one for T cells. The study also detected an increase in IFN-γ and IFN-γ-driven genes in the tumors indicating the presence of Th 1 immunity in the tumor microenvironment [319].

#### Ependymoblastoma

Ependymoblastoma is a rare and highly malignant brain tumor that primarily affects children and significantly impacts their quality of life. Most ependymoblastomas are located in the supratentorial region of the brain, followed by the infratentorial region. In rare cases, ependymoblastomas can be found outside the central nervous system, such as in the sacrococcygeal region, rectovaginal space, or ovaries. The prognosis for ependymoblastomas is very poor. Due to the limited number of case reports and original studies, there are no standardized therapeutic methods for treating ependymoblastoma [320, 321].

Interestingly, in a C57BL/6J mouse model of ependymoblastoma, infection with *T. gondii* resulted in a significant reduction in tumor growth. The infected mice showed a strong inflammatory response surrounding the tumor growth with more necrosis per unit area compared to the control mice. Furthermore, peritoneal macrophages from *Toxoplasma*-infected mice were found to be cytotoxic to ependymoblastoma cells in vitro, while those from control mice were not [322].

#### Neuroblastoma

Neuroblastoma is the most frequently diagnosed cancer in children during the first year of life. It is an embryonic autonomic nervous system tumor usually found in the adrenal glands but can also occur in other areas, such as the head, chest, neck, abdomen, and spine. Treatment options include observation, surgery, radiation, chemotherapy, immunotherapy, or stem cell transplantation [323, 324].

Several studies have shown that certain parasite derivatives can inhibit the growth of tumor cells by stimulating the immune response. Interestingly, Eligio García et al. [217] discovered the presence of antigenic proteins of *T. cruzi* shared with neuroblastoma cells. By confocal microscopy assay, the presence of antigenic proteins by immunodetection in neuroblastoma cells reacted with antibodies against *T. cruzi* suggesting, a cross-reactive immune response [217]. Such finding has valuable implications for the discovery of novel molecules of parasitic origin targeting oncogenic cells.

#### Thymoma

Thymoma is a rare neoplasm that develops from the epithelial cells of the thymus. It makes up 20% of tumors in the mediastinal and is the most common tumor in the anterior mediastinum. Although it usually has an indolent growth pattern, thymoma can invade nearby tissues with intrathoracic recurrences frequency. It is often linked to various immune and non-immune-related paraneoplastic syndromes. The main treatment for thymoma is surgery to completely remove the tumor. Inoperable patients receive chemotherapy first followed by reassessment for surgery, and radiation therapy may also be recommended. New treatment approaches are currently being explored [325].

Preliminary promising results from experimental studies have shown that *N. caninum*, a non-pathogenic protozoan parasite in humans, could be a promising candidate to enrich the repository of anti-cancer immunotherapies. In an in vitro study *N. caninum* (NC1 strain) tachyzoites successfully infected and multiplied inside murine thymoma cells (cell line EG7) and caused their lysis. In a mouse model wild-type murine thymoma EG7, treatment via subcutaneous injection of *N. caninum* tachyzoites either in or far from the tumor resulted in strong anti-tumor immunity and significant inhibition of tumor growth, often leading to complete eradication. Analysis of immune responses revealed that *N. caninum* could destroy infected cancer cells, reactivate the suppressed immune cells, and activate the systemic immune system by generating a protective anti-tumor response dependent on NK cells, CD8+ T cells and associated with strong IFN-γ secretion in the tumor microenvironment. Additionally, modifying *N. caninum* to secrete the IL-15/IL-15Rα in the microenvironment significantly enhanced its immunological properties.

In the same study, the authors compared the anti-tumor capacity of *N. caninum* to that of *T. gondii* (RH strain), a naturally infectious parasite of rodents and humans known to have anti-tumor activity. Intra-tumoral inoculation of *T. gondii* tachyzoites induced a strong inhibition of tumor development and complete eradication of tumors in some mice 25 days post-treatment. Therefore, both the apicomplexan protozoan parasites, *N. caninum* and *T. gondii*, have similar anti-tumor properties against murine thymoma EG7 tumor regardless of whether the mice are a natural or non-natural host of the parasite used. Interestingly, after 18 days post-treatment, *N. caninum* was completely cleared from the tumor, spleen, brain, and liver, indicating a natural clearance of *N. caninum* by the mouse immune system. In contrast, *T. gondii* was still detected in the tumor 25 days post-treatment, and its quantity was 200-fold the initial injected dose, indicating its high ability to multiply and persist compared to *N. caninum*, despite similar anti-tumor efficacy.

These results further support the potential of using a non-naturally infectious agent as a therapeutic due to its strong anti-tumor properties and natural clearance, highlighting its safety. Thus, *N. caninum* could be used as an oncolytic protozoan and engineered strains of *N. caninum* have the potential to be used to either secrete anti-tumor effectors or express proteins at its surface, expanding the potential clinical applications of *N. caninum* to a wide range of cancer therapies [294].

#### Head and neck squamous cell carcinoma

Head and neck squamous cell carcinoma refers to cancers that typically originate from the mucosal epithelium of the oral cavity, pharynx, and larynx. Risk factors for oral cavity and larynx cancers include tobacco use, alcohol abuse, or both, while pharynx cancers are increasingly linked to human papillomavirus (HPV) infection, particularly HPV-16. Treatment usually includes surgery, chemoradiotherapy, EGFR monoclonal antibody, and immune checkpoint inhibitors. Current efforts are focused on identifying more effective and less toxic therapies [326].

Interestingly, Parker and colleagues studied the effect of *T. vaginalis* purine nucleoside phosphorylase (*Tv*PNP), an enzyme for the activation of deoxyadenosine analogs. In human head and neck squamous cell carcinoma, (FaDu) xenografts expressing *Tv*PNP (FaDu-*Tv*PNP) in nude mice, exhibited robust tumor regressions. *Tv*PNP was found to be more efficient than *Ec*PNP for activating deoxyadenosine analogs [316].

Therefore, parasites should be thoroughly explored as the upcoming era of anti-neoplastic agents. Advancements in technology can help address challenges, such as standardizing parasite components, optimizing their delivery methods, and elucidating their mechanisms of action. Assessment of the safety profiles of these novel therapies and advancing promising candidates to clinical trials is a noteworthy step in developing novel, nature-based cancer therapeutics that can revive hope for cancer therapy.

## Conclusion

The aforementioned data highlight the promising anti-neoplastic activity of certain parasites and their derivatives against various cancers. Parasites hold promise to contribute to the development of advanced cancer treatments, including immunotherapeutics as highly immunogenic cancer vaccines, therapeutic antibodies, and immunomodulators, among others. They also offer opportunities for gene therapy, targeted therapy, synergistic combination therapies, and combating drug resistance. Thus, funding agencies must support multidisciplinary research projects involving different scientific disciplines in collaboration with the pharmaceutical industry to accelerate this process to fight cancer.

## Ethics approval and consent to participate

Not applicable.

## Consent for publication

Not applicable.

## Data Availability

No datasets were generated or analyzed during the current study.
